# Encoding of Naturalistic Stimuli by Local Field Potential Spectra in Networks of Excitatory and Inhibitory Neurons

**DOI:** 10.1371/journal.pcbi.1000239

**Published:** 2008-12-12

**Authors:** Alberto Mazzoni, Stefano Panzeri, Nikos K. Logothetis, Nicolas Brunel

**Affiliations:** 1 Division of Statistical Physics, Institute for Scientific Interchange, Turin, Italy; 2 Robotics, Brain and Cognitive Sciences Department, Italian Institute of Technology, Genoa, Italy; 3 Faculty of Life Sciences, University of Manchester, Manchester, United Kingdom; 4 Max Planck Institute for Biological Cybernetics, Tübingen, Germany; 5 Division of Imaging Science and Biomedical Engineering, University of Manchester, Manchester, United Kingdom; 6 Laboratory of Neurophysics and Physiology, Université Paris Descartes, Paris, France; 7 CNRS UMR 8119, Paris, France; University College London, United Kingdom

## Abstract

Recordings of local field potentials (LFPs) reveal that the sensory cortex displays rhythmic activity and fluctuations over a wide range of frequencies and amplitudes. Yet, the role of this kind of activity in encoding sensory information remains largely unknown. To understand the rules of translation between the structure of sensory stimuli and the fluctuations of cortical responses, we simulated a sparsely connected network of excitatory and inhibitory neurons modeling a local cortical population, and we determined how the LFPs generated by the network encode information about input stimuli. We first considered simple static and periodic stimuli and then naturalistic input stimuli based on electrophysiological recordings from the thalamus of anesthetized monkeys watching natural movie scenes. We found that the simulated network produced stimulus-related LFP changes that were in striking agreement with the LFPs obtained from the primary visual cortex. Moreover, our results demonstrate that the network encoded static input spike rates into gamma-range oscillations generated by inhibitory–excitatory neural interactions and encoded slow dynamic features of the input into slow LFP fluctuations mediated by stimulus–neural interactions. The model cortical network processed dynamic stimuli with naturalistic temporal structure by using low and high response frequencies as independent communication channels, again in agreement with recent reports from visual cortex responses to naturalistic movies. One potential function of this frequency decomposition into independent information channels operated by the cortical network may be that of enhancing the capacity of the cortical column to encode our complex sensory environment.

## Author Summary

The brain displays rhythmic activity in almost all areas and over a wide range of frequencies and amplitudes. However, the role of these rhythms in the processing of sensory information is still unclear. To study the interplay between visual stimuli and ongoing oscillations in the brain, we developed a model of a local circuit of the visual cortex. We injected into the network the signal recorded in the thalamus of an anesthetized monkey watching a movie, to mimic the effect of a naturalistic stimulus arriving at the visual cortex. Our results are in striking agreement with recordings from the visual cortex. Furthermore, through manipulations of the signal and information analysis, we found that two specific frequency bands of the neurons' activity are used to encode independent stimuli features. These results describe how sensory stimuli can modulate frequency and amplitude of ongoing neural activity and how these modulations can be used to convey sensory information through the different layers of the brain.

## Introduction

Oscillations are a common and prominent feature of cortical sensory-evoked activity. Presentation of sensory stimuli elicits oscillations in Electro-Encephalogram (EEG) and Local Field Potential (LFP) recordings which span a very broad frequency spectrum, ranging from a fraction of a Hz to well over 100 Hz. Oscillations in the gamma band (30–100 Hz) have elicited a great deal of attention because they are robustly triggered and modulated by sensory stimuli in olfactory [1], auditory [2],[3] and visual cortices [4]–[9]. In addition, particular types of behaviorally relevant stimuli (such as stimuli with either rhythmic, complex, or naturalistic dynamics) elicit and modulate cortical oscillations at specific frequencies within the low-frequency (<10–20 Hz) range [10]–[16]. The prominent presence of oscillations in sensory systems raises two sets of important questions: how are these oscillations generated? and why are they generated? In other words, what is the mechanism of the oscillations, and what is their function?

The first question has motivated many recent theoretical studies. Theorists have proposed different mechanisms giving rise to oscillatory activity in models of recurrent networks of spiking neurons. In networks coupled through purely chemical synapses, oscillatory synchrony might emerge through mutual inhibitory interactions [17],[18], or due to a feedback loop between excitatory and inhibitory neurons [19],[20]. Recent studies have focused on a regime of high noise, due to the observed irregularity of firing of neurons in cortex [21]–[24]. These studies have demonstrated the existence of an oscillatory regime in which a population of cells fire rhythmically at high frequencies, while single cells fire stochastically at a rate that is much lower than the population frequency. The network frequency was shown to depend on synaptic time scales [25],[26], as well as on the balance between excitation and inhibition [26],[27]. In a large parameter range, the network frequency is in the gamma range [26],[27]. One of the interesting features of this oscillatory regime is that it strongly depends on external inputs. For weak external inputs, the network is typically in an asynchronous state, with small damped oscillations due to finite size effects [25]. As the inputs increase, the network becomes more synchronized, and the amplitude of the oscillation increases.

In spite of the effort to understand the mechanism of generation of network oscillations, the role of such oscillations in information encoding has remained so far elusive, and several key questions have yet to be addressed. First, there is currently no theoretical framework that explains how, even in the same sensory area, different types of stimuli encode information in different frequency bands [8],[10],[14]. Second, although there is evidence that external stimuli with a rhythmic structure may entrain low frequency cortical oscillations [10],[16], it is not known how the combination of fluctuations generated by stimulus-neural interactions and the oscillations generated by neural-neural interactions shapes the network dynamics and serves sensory information encoding. Third, the potential computational advantages of the cortical encoding of stimuli by a diverse and broad range of frequencies have not been understood yet.

Here, we hypothesize that stimulus-related changes of low-frequency cortical fluctuations originate directly from stimulus-neural interaction and encode information about slowly varying features in the sensory or thalamic input, whereas the stimulus-related changes of high-frequency cortical oscillations are mediated by neural-neural interactions and carry information about sensory features that provoke thalamic responses that differ only in terms of their total spike rate. We tested this hypothesis by simulating a network of excitatory and inhibitory neurons modeling a local population in primary visual cortex, and we determined how the LFPs and spiking activity generated by the network encode information about either simple or complex inputs, the latter simulating sensory-related thalamic signals. We found that the simulated network encodes dynamic stimulus features according to the hypothesis described above, and in particular encodes naturalistic stimuli by using low and high response frequencies as independent communication channels, in agreement with results from visual cortex [14].

## Results

**Figure 1:**
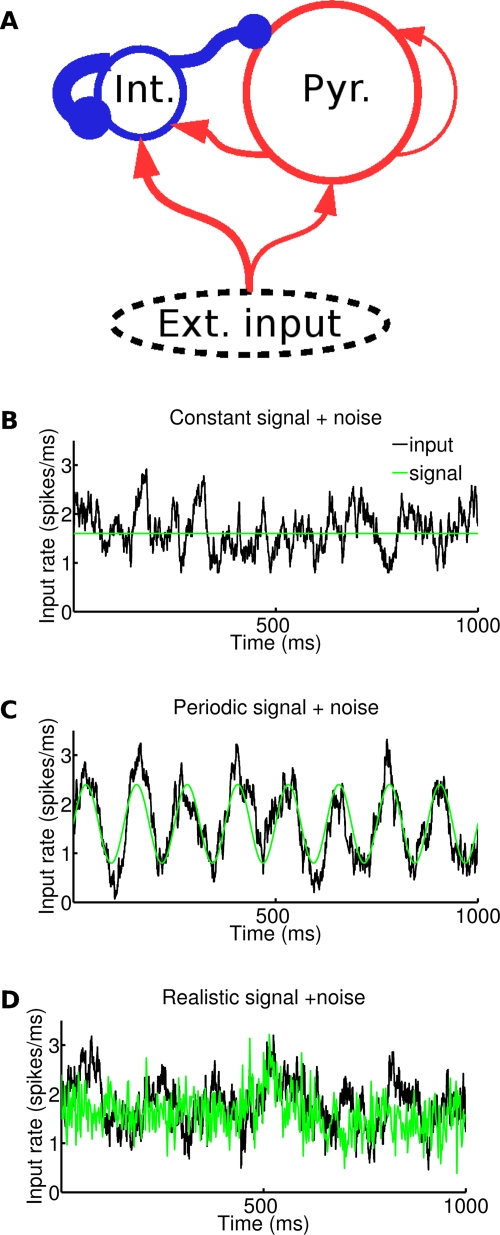
Network structure and inputs. (A) The network is composed of two populations (1000 interneurons and 4000 pyramidal neurons). The connectivity is random, a synapse being present between any directed pair of neurons with probability 0.2. The size of the arrows represents schematically the strength of single synapses: recurrent interactions are dominated by inhibition. In addition to recurrent interactions, both populations receive an external excitatory input. (B–D) Three types of inputs are delivered to the network. The three panels display (in black) the time-varying rate of Poissonian spike trains representing external inputs to each neuron in the network in a 1 second long interval. All inputs are a superposition of a ‘signal’ and a ‘noise’ component. The ‘signal’ is shown in green. Average value of input is 1.6 spikes/ms in all traces. The noise is modelled as an Ornstein-Uhlenbeck process (see Methods) in all cases while the three signals are different, (B) Signal: constant rate. (C) Signal: oscillatory rate (here shown with 8 Hz frequency and 0.8 spikes/ms amplitude) (D) Signal: taken from MUA recordings of LGN of anesthetized monkeys watching natural movie scenes (see Methods).

We used a model of cortical network composed of leaky integrate-and-fire neurons, similar to the one used in [26] (Figure 1A). In brief (see Methods for full details), the model network represents in a simplified way a local circuit in primary visual cortex, and was composed of two neuronal populations: 1000 inhibitory interneurons and 4000 pyramidal neurons. The network connectivity was random and sparse with a 0.2 probability of directed connection between any pair of neurons. Synaptic currents represented fast synaptic interactions, with time courses resembling experimentally measured AMPA currents (for excitatory currents) and GABA currents (for inhibitory currents). The strength of GABAergic connections was sufficient to ensure stable activity at low firing rates in the network. Both populations received a noisy excitatory external input taken to represent the activity from thalamocortical afferents, with interneurons receiving stronger inputs than pyramidal neurons [28].

We quantified the network activity by monitoring the individual spike times of each neuron, the instantaneous population firing rate (obtained counting the number of spikes fired by neurons in a given population in a 1 ms bin), the average membrane potential of each population, and the average synaptic currents. Since the spiking activity of individual cortical neurons is irregular, oscillations are often monitored experimentally by recording LFPs. Thus, to better compare the oscillations of our model to those recorded in cortex, we computed a simulated LFP from our network (see Methods for a complete description).

LFPs are experimentally obtained by low pass filtering the extracellularly recorded neural signal, and are thought to reflect primarily the current flow due to synaptic activity around the tip of the recording electrode [29], as well as some other type of slow activity such as voltage-dependent membrane oscillations [30] and spike afterpotentials [31]. Thus, we computed the LFP as the sum of the absolute values of AMPA currents and of GABA currents. Since pyramidal neurons contribute maximally to generation of LFPs in cortex because their apical dendrites are organized in an approximate open field configuration, we summed only currents from synapses of the pyramidal neurons population. This model, though much simpler than some previous models [32], proved to be an effective way to generate a realistic LFP signal that match many characteristics of LFPs in sensory cortex, as shown below.

**Figure 2:**
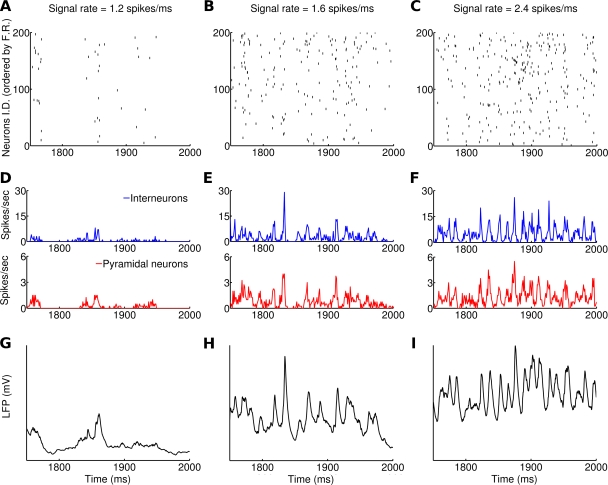
Dynamics of the network receiving a constant signal, with three different rates (left, middle, right column: 1.2, 1.6, 2.4 spikes/ms), superimposed to noise. In each column, all panels show the same 250 ms interval (extracted from a 2 seconds simulation). (A–C) Raster plot of the activity of 200 pyramidal neurons (those that had the highest firing rate during the simulation). (D–F) Average instantaneous firing rate (computed on a 1 ms bin) of interneurons (blue, upper panels) and pyramidal neurons (red, lower panels). Notice the difference in scale. (G–I) LFP of the network, modeled as the sum of the absolute values of AMPA and GABA currents on pyramidal neurons (see Methods). Notice that the population oscillations become more pronounced as the rate of the signal increased, while oscillations are not detectable at the single neuron level.

As a consequence of strong recurrent inhibition, single neurons fire in an irregular fashion at low rates [25], [33]–[37]; however population oscillations are clearly visible at the network level [25],[26] (as shown in Figure 2). Since they were present when the input to the network was constant in time, these oscillations must be generated within the network. As demonstrated previously, two features of the recurrent connectivity contribute to the generation of network oscillations: delayed interactions between interneurons, which tend to favor high frequency oscillations [25],[26], and the excitatory-inhibitory feedback loops, that tend to promote lower frequency oscillations [26]. The oscillation frequency depends on delays, synaptic time constants and the balance between excitation and inhbition [26]. For the model parameters chosen here (see Methods), the frequency of the oscillation was in the 30–100 Hz range, similar to experimental observations [14].

One crucial property of such excitatory-inhibitory recurrent networks is that the strength of the population oscillation strongly depends on external inputs to the network. Typically, for low enough external inputs, the network is in an asynchronous state, with weak and strongly damped oscillations in the population activity due to finite size effects, while for strong external inputs, the network tends to settle in a pronounced oscillatory state [25],[26],[37]. The goal of the present paper is to analyze how the modulation of this internally generated oscillatory synchrony and the interaction between stimulus oscillations and neural oscillations allow the population activity to transmit information about the signals received by the network, and to compare the results with available experimental data [8],[14] in order to better understand the transformation between stimuli and cortical oscillatory activity. To study how stimuli modulate the activity of the model cortical network, we injected to the network three classes of inputs of increasing complexity (Figure 1B–D), composed by different kinds of signals to which we superimposed a noise (see Methods) that was different from simulated trial to simulated trial. We first considered inputs that are constant in time and vary only in rate; we then considered periodic inputs of different frequency and amplitude, and we finally considered complex broadband inputs with a statistics similar to that of geniculate neurons responding to naturalistic movies.

### How Gamma Oscillations Are Modulated by the Firing Rate of the Input Stimulus

We started by examining the network response to 2 seconds long constant signals with different rates, superimposed to noise (Figure 1B). Figure 2 illustrates the dynamics of the system for different rates of the signal (1.2, 1.6 and 2.4 spikes/ms). Raster plots in Figure 2A–C show that the neuronal firing was sparse in all conditions: the average firing rate of individual pyramidal neurons was 0.19, 0.45, 0.92 spikes/sec respectively, whereas the average firing rate of individual interneurons was 0.75, 1.76 and 3.95 spikes/sec respectively. Though spiking activity of single cells was seemingly random, inspection of the total firing rate from the pyramidal and the interneuronal population (Figure 2D–F) showed that increasing the signal rate led to an increased average firing in the network and that population spikes occurred in a synchronous fashion, due to pronounced population oscillations [26] in the gamma band (30–100 Hz). The increase of gamma oscillations with stimulus rate was also clearly visible in the simulated LFPs displayed in Figure 2G–I.

**Figure 3:**
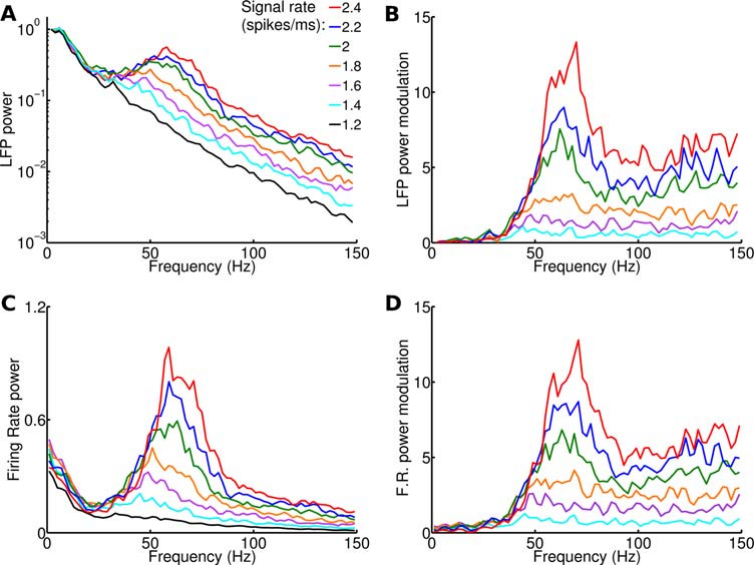
LFP and firing rate power spectrum as a function of signal rate. Each stimulus was composed of a constant signal with a given rate (indicated in the legend) plus noise. Power spectra are averaged from 20 trials of 2 seconds each with different noise realizations. Color code is the same for all panels. (A) LFP power spectrum for various signal rates. (B) Modulation of LFP spectrum for various signal rates. Modulation is defined as the difference of the power of a frequency at a given signal rate and its power at 1.2 spikes/ms signal rate, normalized to the latter power. Compare with Figures 2 and 4 of [8] (C–D) Same as (A–B) for firing rate spectrum. Notice the difference in scale between (A) and (C).

The trial-averaged power spectra of LFPs measured in response to a wide range of firing rate values of the signal are displayed in Figure 3A. The simulated LFPs obtained in responses to such stimuli are of interest because they can be compared directly to the cortical LFPs recorded experimentally in V1 of anesthetized monkeys in response to grating stimuli with different levels of contrast and reported in Ref [8]. This is because the increase in contrast in visual stimuli leads to an increase of average firing rate in LGN [38],[39]. Consistent with the results of [8], we found that the spectra of the simulated LFP showed the highest power at low frequencies, with a local peak in the gamma range (30–100 Hz). The height and the width of the gamma range spectral peak increased monotonically with the signal firing rate. To better visualize how signal rate modulates different frequency bands of the LFP, we defined (in analogy with [8]) the power modulation at given frequency and signal rate as the difference between the trial-averaged spectral power at that frequency in response to the considered input rate and the trial-averaged spectral power at that frequency in response to the smallest input rate tested (1.2 spikes/ms), normalized to the latter power. Modulation values are reported in Figure 3B. Frequencies below 30 Hz in the simulated LFP spectra were only weakly modulated. Frequencies that were more strongly modulated by the stimulus were in the gamma band, with a peak at ∼70 Hz. The modulation reached a plateau at higher frequencies (>100 Hz). All these results are fully consistent with the neurophysiological experiments reported in [8] (see their Figures 2 and 4), which report a strong modulation of the LFP power by visual contrast in the gamma band (30–100 Hz), a smaller modulation at higher frequencies, and very weak modulations at lower frequencies.

**Figure 4:**
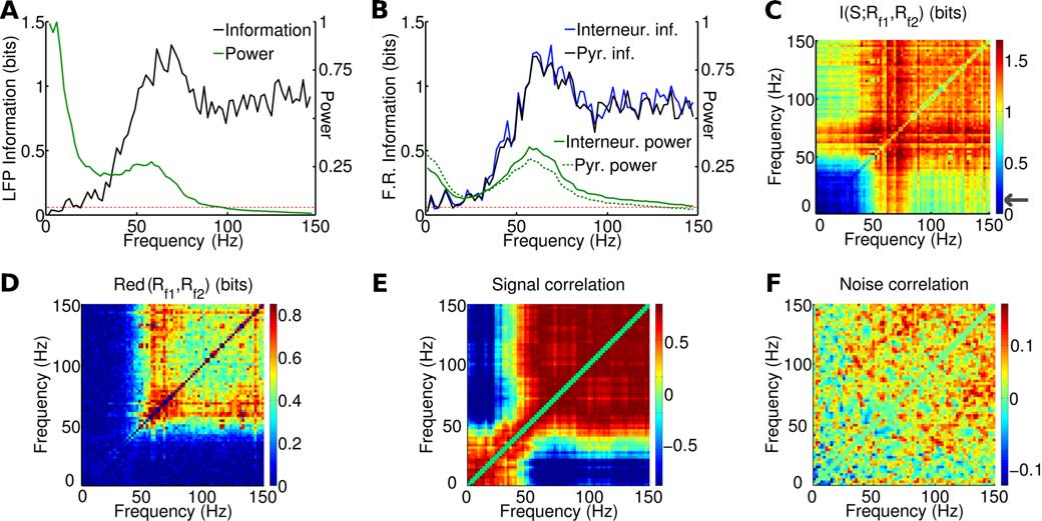
Information content of LFP and firing rate power spectrum relative to constant stimuli with different rates. Each stimulus was composed by noise plus a constant signal with a rate ranging from 1.2 to 2.6 spikes/ms, and presented 20 times for 2 seconds with different noise realizations. (A) Information content of LFP spectrum (in black). The power spectrum averaged over all signals and trials is displayed in a linear scale with arbitrary units for comparison (in green). Red dashed line corresponds to significance threshold (p<0.05; bootstrap test) for information. (B) Information content of the spectrum of the pyramidal (black) and interneurons (blue) population firing rates. Power spectra are displayed with dashed and continuous green line, respectively. Red dashed line as in (A). (C–F) Analysis of LFP frequency pairs: (C) joint information, i.e. information obtained by considering the two frequencies of the pair (see Equation 13). The gray arrow in the color scale indicates significance threshold (p<0.05, bootstrap test). (D) Redundancy, i.e. the difference between the sum of the two information contents and the joint information. (E) Signal correlation, i.e. the correlation across stimuli of trial averaged responses. (F) Noise correlation, i.e. the correlation for fixed stimulus of fluctuations across trials.

We next examined the behavior of the total firing rate. Unsurprisingly, the power spectra of the instantaneous population firing rate varied with signal rate in a way which was very similar to the LFP (Figure 3C and 3D). The relative weight of gamma oscillations was stronger in the interneuron population than in pyramidal neurons; nonetheless the size of the modulation in the gamma band was very similar (Figure 4B).

The above power modulation analysis reveals the frequencies at which the trial-averaged power is most modulated by the stimulus, but it does not tell how easy it is to gain information about the stimulus by observing the LFP in a single trial. To address single-trial discriminability, we used Shannon information (defined in Methods and abbreviated as “information” in the following). We delivered to the network constant signals with 8 different rates ranging from 1.2 to 2.6 spikes/ms. Each stimulation lasted 2 seconds and was repeated for 20 trials, with noise generated independently from trial to trial (see Methods). From these simulated responses, we computed the information *I*(*S*; *R_f_*) between the LFP power *R_f_* at a given frequency *f* and the stimulus input rate *S*. The information *I*(*S*; *R_f_*) is plotted as a function of frequency *f* in Figure 4A and 4B. LFP information was very small at frequencies below 30 Hz, and was high within the gamma range, where it reached a peak of 1.32 bits at a frequency of 70 Hz (to be compared with a stimulus entropy of 3 bits). Information then decreased to an average value of 0.85 bits at higher LFP frequencies (>100 Hz). It is interesting to note that the information peak was reached at a higher frequency than the one at which gamma-range oscillation power was highest. This is consistent with the empirical observation of [7] and can be explained by the fact that the power is maximally modulated by the stimulus at frequencies higher than the peak, since when the input rate is increased the gamma peak is at the same time increasing in power and moving toward higher frequencies (see Figure 3A and 3C).

After determining which LFP frequencies convey the most information about the stimulus, the next step is to investigate whether the information carried by different frequencies is redundant or independent. This can be done by computing the redundancy, defined as the difference between the sum of the information provided by each individual frequency *I*(*S*; *R_f_*_1_)+*I*(*S*; *R_f_*_2_) and the joint information *I*(*S*; *R_f_*_1_ *R_f_*_2_) carried by the joint observation of power at frequency *f*1 and *f*2 (see Equation 14). Results are reported in Figure 4C and 4D. For any frequency above 50 Hz, the joint information *I*(*S*; *R_f_*_1_ *R_f_*_2_) is on average 0.51 bits less than *I*(*S*; *R_f_*_1_)+*I*(*S*; *R_f_*_2_), and redundancy is highly positive: on average for this range the 60% of the information of the less informative frequency in the pair. Thus, the gamma-range representation of the input spike rate is highly redundant.

Information redundancy can happen because the two frequencies are tuned in the same way to the stimulus features, or because they share correlated sources of noise. The correlation of the mean responses across different stimuli of two frequencies are called “signal correlations” [40],[41] because they are entirely attributable to stimulus selectivity. Correlations manifested as covariations of the trial-by-trial fluctuation around the mean response to the stimulus are traditionally called “noise correlations” [40],[41]. Since these noise covariations are measured at fixed stimulus, they ignore covariations effects attributable only to shared stimulation.

To understand better the causes of redundancy, we therefore computed the amount of signal and noise correlation. Figure 4E reports the amount of signal correlation between frequencies *f*1 and *f*2 (computed, for each frequency pair, as the Pearson correlation across stimuli of the trial-averaged responses). Signal correlation was very high across all gamma frequencies (mean in the gamma range: 0.71), showing that all frequencies were tuned to the same stimuli. This is consistent with the above finding (Figure 3) that the height and the width of the gamma range spectral peak increased monotonically with the input firing rate. Since the presence of signal correlations always decreases the joint information and leads to redundancy, this explains the redundancy between frequencies. Figure 4F reports the amount of noise correlation (computed as the Pearson correlation coefficient across trials at fixed stimulus of the trial-average-subtracted powers at frequency *f*1 and *f*2, averaged over all stimulus windows). There was little noise correlation (mean in the gamma range: 0.05), which means that redundancy is due to signal correlation.

**Figure 5:**
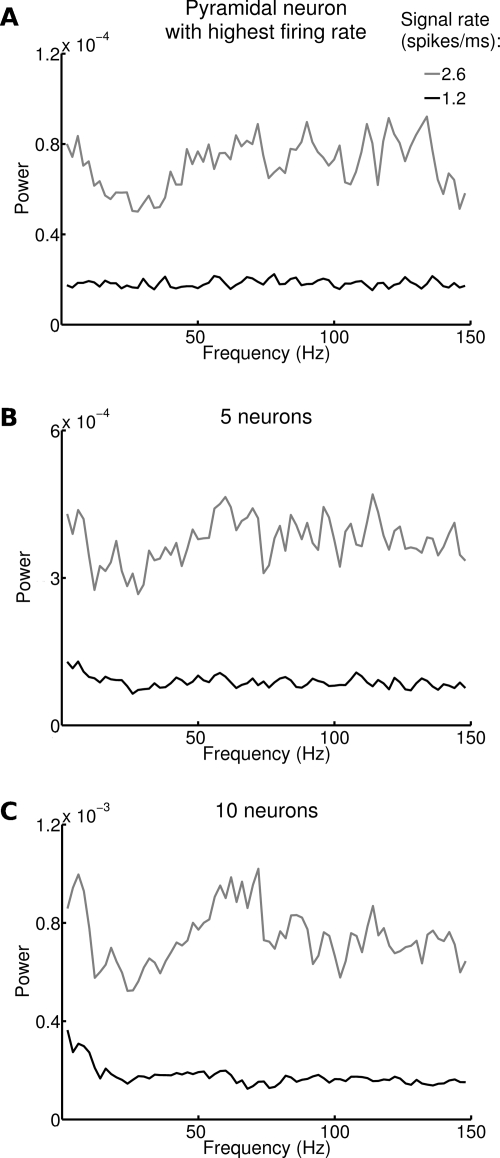
Spectral modulations associated to changes in stimulus rate in ensembles of a small number of neurons. (A) Power spectrum of firing of pyramidal neuron with highest firing rate when the signal rate is 1.2 and 2.6 spikes/ms. Averages over 20 trials displayed in black and gray, respectively. (B–C) Same as (A) for the total firing rate of the 5 and 10 pyramidal neurons with highest firing rates, respectively. Notice the difference in the power scale.

In experimental conditions, it is typically possible to record spikes from a limited set of neurons, and not from all neurons in a local network. Since single neurons fire irregularly at rates which are much lower than gamma frequencies, it is essentially impossible to detect the gamma oscillation from single neuron spike trains. What is the minimum amount of neurons necessary to detect gamma band modulations driven by the stimulus rate? We address this question in Figure 5, where we plot the power spectrum of the average activity of ensembles of a small number of neurons (from 1 to 10), for two different input signal rates. We have considered only pyramidal neurons for this analysis because they have a larger soma, so they are more likely to be recorded extracellularly and offer hence a clearer comparison with experimental results. Figure 5A shows the power spectrum of the pyramidal neuron with highest average firing rate when the signal rate was varied from 1.2 to 2.6 spikes/ms. In both cases the power spectrum was flat, consistent with the neuron firing approximately as a Poisson process in all stimuli conditions. The only effect of changes in stimulus rate was to increase the power uniformly at all frequencies, proportionally to the mean firing rate (maximum firing rate of 10.6 spikes/sec). Pooling together the spikes of the 5 neurons with highest firing rates was still not enough to detect gamma oscillations (Figure 5B). It was necessary to pool together the 10 neurons with highest firing rates, to see a clear peak in the gamma band for the highest rate stimulations (Figure 5C). In this case, the compound firing rate of all pyramidal neurons in the set reached 85 spikes/sec.

### Response of the Network to Low Frequency Oscillatory Inputs

**Figure 6:**
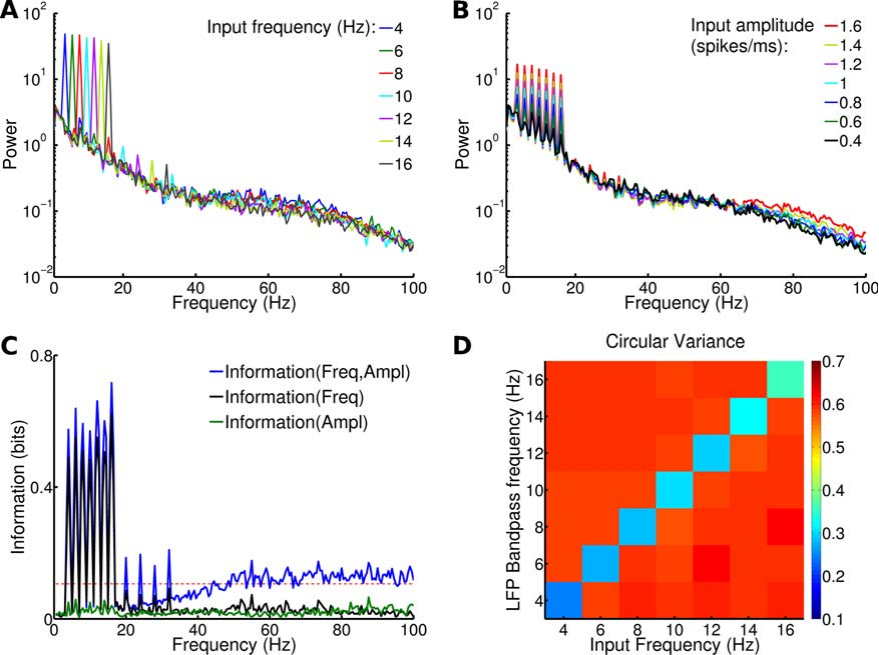
Modulations in the power spectrum of LFP due to changes in the spectral content of the input. Each stimulus was composed by noise plus a periodic signal. Signal amplitude *A* varied from 0.4 to 1.6 spikes/ms and signal frequency *ω* from 4 to 16 Hz. Modulations were studied (i) across the whole range of stimuli, (ii) pooling together all the responses to stimuli with the same frequency, (iii) pooling together all the responses to stimuli with the same amplitude. (A) LFP power spectra across set (ii). Data are averaged over 20 trials and over the set of amplitudes. (B) LFP power spectra across set iii). Data shown are averaged over 20 trials and over the set of frequencies. (C) Information associated to changes in stimulus spectral content. Information relative to set (i), (ii) and (iii) is respectively displayed in blue, black and green. Red dashed line corresponds to significance threshold (p<0.05; bootstrap test) for information. (D) Circular variance of phase difference between the input signal and the LFP bandpassed at different frequencies (with a 2 Hz range). The circular variance was averaged over all trials and amplitudes.

Most previous model studies of oscillations in cortical networks consider, as we did above, the network dynamics in response to time-independent input stimulation. However, naturalistic stimuli are not static, but vary on time scales which are typically much slower than the time scales of network oscillations discussed above [42]. As a preliminary to the study of the network dynamics under natural stimulation conditions, we thus next examine the network dynamics in response to periodic input signals that oscillate at frequencies below 20 Hz. We stimulated the network with periodic signals (see Methods) characterized by their amplitude *A* (7 different amplitude values, ranging from 0.4 to 1.6 spikes/ms in 0.2 spikes/ms steps), and their frequency *ω* (7 different frequency values, ranging from 4 to 16 Hz in 2 Hz steps). Each signal was presented to the network superimposed to noise (Figure 1C) in 20 different trials, each one lasting for 2 seconds. Figure 6A shows the trial-averaged LFP spectra for different frequencies *ω* of the input signal, averaged over all presented amplitudes *A*. Input oscillations in this low frequency range are reproduced in the LFP, causing a peak in the spectrum at exactly the input frequency, with little effect on the rest of the spectrum, apart from a very small modulation of the power of the gamma range. This suggests that different low frequencies in the stimulus are represented by the LFP independently from each other and are almost entirely encoded as entrainment of the corresponding low-frequency LFP band. Figure 6B reports the trial-averaged LFP spectra for different signal amplitudes, averaged over all presented frequencies. The LFP spectral peaks originated by the different low frequencies in the input did not shift place when the amplitude was increased and only increased the height of their peak, again compatibly with an entrainment with the stimulus. When the amplitude of the oscillation was increased from 0.4 to 1.6 spikes/ms, the peak power corresponding to the input frequency increased linearly of a factor 4.7±0.4 for all considered frequencies. Very similar results were obtained analyzing the power spectrum of the total firing rate (data not shown).

To investigate whether the low frequency band was more sensitive to amplitude or frequency modulations, we used again information theory. Figure 6C plots (blue line) the information that the LFP power at frequency *f* conveyed about both the frequency *ω* and the amplitude *A* of the input spike rate. The information contained in the peaks in the signal frequencies range was in between 0.55 and 0.71 bits for a total stimuli entropy of 5.6 bits. The information about *ω* and *A* was significant (p<0.05; bootstrap test) only at the LFP frequencies corresponding to the input ones (with smaller peaks at their first harmonics). However, the LFP at a given frequency may actually represent only a smaller subset of stimulus parameters; for example, either *A* alone or *ω* alone. To reveal which parameter is encoded at each frequency, we used the stimulus grouping approach of [43]. In this approach, stimuli were grouped into classes of frequency or of amplitude. When this was done, the number of unique stimuli in the set was reduced. For frequency grouping, the 49 stimuli defined by joint values of *ω* and *A* were reduced to seven groups in which all stimuli within a group had the identical value of *ω*. Likewise, amplitude grouping yielded seven groups defined by identical values of *A*. Applying the “data processing inequality” [44], it follows that the information about the frequency or amplitude-grouped stimuli must be less than or equal to the information about the full, ungrouped stimulus set made of amplitude and frequencies. Grouped and ungrouped information can be equal if, and only if, the LFP power responds only to the stimulus feature that characterizes the grouped responses [44]. We computed the grouped information carried by the LFP at frequency *f* about either *A* only (green line) or *ω* only (black line). The information about *ω* conveyed by low frequency LFPs was larger than the one conveyed about *A*. This means that low LFP frequencies are more sensitive to modulations in the signal frequency than in the signal amplitude.

To characterize entrainment of network activity by the input signal, we measured the circular variance of the phase difference between the signals and the band-passed LFP (see Methods). The value of this measure ranges from zero (signal and LFP are perfectly phase locked for a given frequency window), to one (the phase difference changes randomly). Figure 6D displays the average of the circular variance over all trials and amplitudes and shows that periodic stimuli were able to entrain the LFP at the corresponding frequency. The effect was stronger for lower frequencies and for larger signal amplitudes (Figure S1).

### LFP Responses to Complex Input Stimuli with Naturalistic Dynamics

The results obtained so far with constant and periodic input signals suggest that low LFP frequencies contain information about the corresponding low frequencies in the input signal, while gamma LFP frequencies contain information mostly about the spike rate of the input stimulation. To understand the implications of these coding rules, we now turn to the study of the LFP responses to inputs with a broadband naturalistic temporal structure.

We aimed at simulating responses of visual cortex dynamics during the viewing of naturalistic movie stimuli, for which detailed neurophysiological data of LFP cortical responses are available [14],[15]. We thus built a naturalistic input that closely matched the time course of multiple-unit activity (MUA) recorded from LGN of anesthetized monkeys that were presented with natural color movies (see Methods for details).

**Figure 7:**
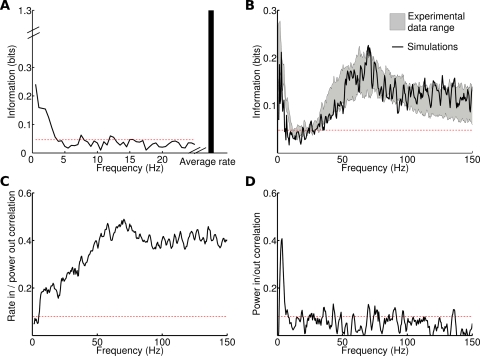
Information content relative to naturalistic stimuli, based on MUA recordings from LGN of an anesthetized monkey watching natural movie scenes. Recording time (40 seconds) was divided into 20 intervals, considered as different signals. Each signal was injected 20 times with different noise realizations (see Methods). Red dashed horizontal line indicates significance threshold (p<0.05; bootstrap test) in all panels. (A) Information content of different frequencies and average rate of naturalistic input. (B) Information content, relative to naturalistic inputs, of simulated LFP (in black) compared with the information content of LFP recorded in V1 in the same experiment from which LGN data were taken. Gray area represents the *mean*±*std* range of information across 7 different electrodes recording synchronously from different sites. (C–D) Correlation between LFP spectrum and signal features. (C) Correlation between stimulus rate and power of LFP frequencies in the response. (D) Correlation between the power of each frequency in the stimulus spectrum, and its power in the corresponding LFP spectrum.

We started by analyzing how different frequencies of the LGN MUA signal encode information about which scene of the movie was presented to the animal. This analysis documents the characteristics of the information injected to the network. Figure 7A reports the information that the power of LGN MUA signal described above encodes about the movie scenes; the total entropy of the movie scene characterization was 4.3 bits. Almost all the information in the LGN MUA (which provided the naturalistic input to the simulated network) was carried by the power at frequencies below 5 Hz (peak at 1 Hz with a value 0.24 bits) and in the average spike rate (the DC component of the LGN MUA), carrying 1.27 bits of information. Interestingly, different low frequencies of the LGN MUA signal carried independent information about the visual stimulus: their information redundancy, as well as any noise or signal correlation, was very small: the mean redundancy between frequencies carrying significant information was 0.008 bits (see Figure S2). The average spike rate too conveyed independent information from that carried by modulations at low frequencies: the mean redundancy between frequencies carrying significant information and the spike rate was 0.007 bits.

Once we documented the properties of the naturalistic input, we injected it in the network and we measured the information about the stimuli that was carried by the power of the simulated cortical LFP at each frequency *f* (Figure 7B). There were two frequency regions in which the simulated network LFP was highly informative about the stimuli. The first informative LFP region was in the range 1–5 Hz. The peak information value in this region was 0.21 bits, and was reached for the 3 Hz frequency (Figure 7B). The amount of information contained in the low frequencies of the LFP was similar to the one contained in the same band of the naturalistic input. The second highly informative LFP frequency range was inside the gamma band, in the range of 50–80 Hz (Figure 7B). The peak information value at high frequencies was 0.23 bits. Intermediate simulated LFP frequencies (in the range 6–30 Hz) carried no significant information about the naturalistic input (p>0.05; bootstrap test). It is interesting to compare the information carried by the power of the simulated LFPs to the information carried by real visual cortical LFPs during stimulation with a color movie [14]. Figure 7B compares the information carried by the simulated LFPs with the information carried by real LFPs obtained from seven different electrodes from monkey V1 [14], which were recorded simultaneously with the very same LGN MUA data used to construct the input to the simulated network. The information about the stimuli were computed with exactly the same procedures on both simulated and real data, and are thus directly comparable. Figure 7B shows a very close agreement between simulated and real V1 LFPs.

The agreement between model and data was measured with the reduced *χ* ^2^ (see Methods). The model described correctly both the shape and the information content of the spectrum of the recorded LFP (Table 1). The only appreciable difference between simulated and real data is that the low frequency peak of simulated data decays to a non-significant value at lower frequency (5 Hz) than that of V1 LFPs (whose information drops to a non-significant value at 10 Hz). One potential explanation for this discrepancy is that the input information to our cortical network decayed to zero within 5 Hz, and that low frequency LFPs just follow this trend (see below for an explicit demonstration). However, the LGN MUA signal that we used as input represents only a part of the real inputs to V1, which may receive additional information in the 5–10 Hz frequency range from other sources.

**Table 1:** Goodness-of-fit with experimental data of model LFP in presence of signal and synaptic modulations, measured with 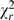 (see Methods).

	Spectrum Information Content	Average Spectrum
Model	0.7	1
Baseline 1	1.9	1.4
Baseline 3	2.1	1.2
Averaged signal	1.5	1.4
GABA 140	0.8	2.8
GABA 60	1.2	150
AMPA 80	8.1	11
AMPA 120	1.7	45

Simulations with periodic and constant signals suggest that the information contained in the low frequency and gamma peaks of Figure 7B corresponds respectively to information about the low frequency modulations of the signal and its rate. To evaluate in detail this hypothesis, we calculated the correlation across all stimuli and trials between the power of each frequency in the LFP spectrum and the average rate of the signal (Figure 7C). As expected, frequencies below 5 Hz are not significantly correlated with the signal rate, while LFP frequencies above 50 Hz are strongly correlated with it (with a peak at 70 Hz). We then calculated the correlation across all stimuli and trials between the power of each frequency in the LFP spectrum and the power associated to the same frequency in the signal spectrum (Figure 7D). Low-frequency LFPs up to 8 Hz were significantly correlated to the signal frequency modulations, while LFP frequencies above 50 Hz were not. Again, very similar results were obtained analyzing the power spectrum of the total firing rate (data not shown).

The entrainment between signal and LFP was measured band-passing both for frequencies ranging from 2 to 15 Hz and then computing for all pairs of signal and LFP frequencies the circular variance of the phase difference (see Methods). The average over all trials and scenes of the phase circular variance was 0.12 between the signal and LFP when they were both bandpassed at frequencies below 4 Hz. It was larger than 0.5 for every other combination of frequencies. Hence, entrainment is restricted to very low frequencies. This could be due to the fact that only low frequency oscillations in the signal are strong enough to override the noise and that entrainment is stronger for low frequency oscillations (see Figure 6D).

Therefore, results obtained with simple stimuli are still valid when stimuli are realistic, suggesting that the two “information channels” (low frequencies and gamma band) could represent the sensory stimuli in a largely independent way.

**Figure 8:**
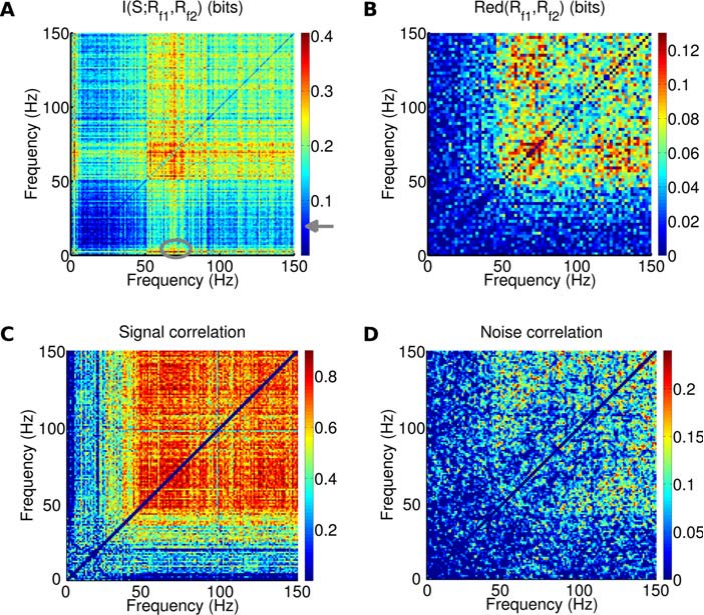
Frequency correlations in LFP when network was presented with naturalistic stimuli. (A) Joint information for frequency pairs. The ellipse indicates the maximum value, obtained for pairs composed by a frequency _<_5 Hz and gamma band frequencies. The arrow indicates significance threshold (p<0.05, bootstrap test). (B–D) Values of (B) Redundancy, (C) Signal correlation, (D) Noise correlation, for frequency pairs in the LFP spectrum. Measures were computed for frequencies set at least 2 Hz apart.

As a step toward gaining a more quantitative insight on how different LFP frequencies encode information about the naturalistic stimulation, we next considered the information 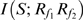 about the stimuli that can be extracted from the joint observation of the powers of two LFP frequencies *f*_1_ and *f*_2_. Figure 8A shows that the highest peak in joint information (0.43 bits) was reached when combining one low frequency (3 Hz) and one gamma range frequency (70 Hz), in nice agreement with results obtained from real V1 LFPs during movie stimulation [14]. The information 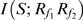 was smaller when both *f*_1_ and *f*_2_ were in the gamma range (<0.4 bits; Figure 8A).

Why is it more convenient to extract information about the naturalistic stimulus by considering one low and one high gamma frequency LFP? Figure 8B explains this finding by considering the redundancy of the information about the stimuli obtained from two different LFP frequencies. Gamma frequencies were all very redundant to each other: frequencies in the 50–80 Hz range shared on average 0.08 bits of redundancy. In contrast, low and high frequencies shared an information redundancy which was close to zero, again in agrement with [14]. This suggests that gamma and low frequencies band contained information about largely independent stimulus features. Also the redundancy between pairs of frequencies below 5 Hz was close to zero, suggesting that each frequency in this range was modulated independently across signals.

Figure 8C reports the signal correlation between pairs of LFP frequencies, which quantifies the similarity in stimulus tuning of the power of LFPs at different frequencies. Signal correlation was very high (up to 0.8) among frequencies in the gamma range, which means that they are all modulated in a similar way by the naturalistic stimuli, and explains why gamma range LFPs convey mutually redundant information about them. However, the signal correlation between the informative low frequency LFPs and the gamma LFPs was negligible, which means that these two frequency ranges are tuned to very different stimulus features. Figure 8D reports the noise correlation between any pair of different LFP frequencies, which measure if trial-to-trial fluctuations around the mean response are correlated. Noise correlation was negligible in the entire frequency range, which implies that the gamma-range redundancy is entirely attributable to signal correlation. Since low and gamma frequency LFPs shared neither noise nor signal correlation, it means that low frequency LFPs and gamma LFPs are completely decoupled in natural stimulation condition, and this is why they add independent information about the stimulus. This result is again fully consistent with the experimental finding of [14]. The only discrepancy between signal and noise correlation in real data [14] and in the present model is that real data presented strong noise correlation within the low frequency LFP range (<24 Hz) [14]. The significance of this discrepancy will be addressed in Discussion.

As a final step to understand the effect of the input characteristics on the network dynamics, we selectively manipulated different features of the signal and quantified the differential effect of these manipulations on the low and high frequency network LFPs.

**Figure 9:**
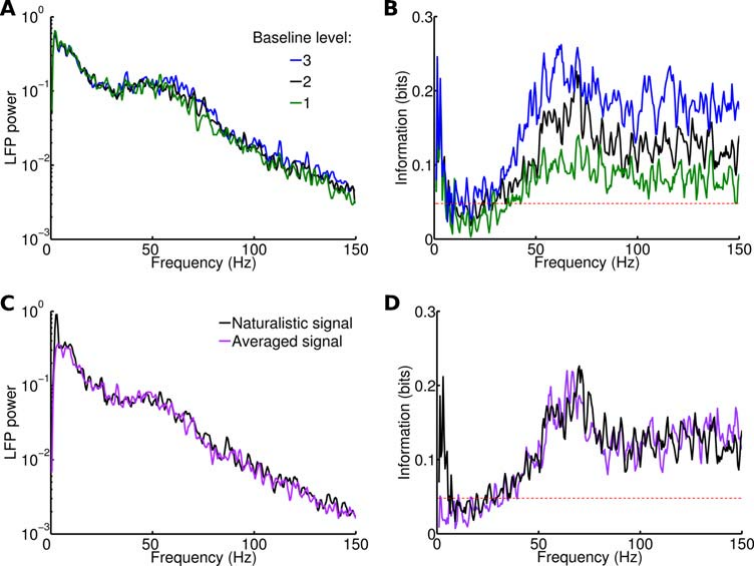
Effects of modulations of naturalistic stimuli. (A) Power spectrum of LFP during a single stimulus, for three different levels of baseline of the same signal. Results averaged over 20 trials. (B) Information content of LFP spectrum for three different levels of baseline for the whole input. Same color code as (A). Red dashed line corresponds to significance threshold (p<0.05; bootstrap test) for information. (C) Power spectrum of LFP during a single stimulus with a naturalistic signal (in black) and with the same signal averaged (in purple). Results averaged over 20 trials. The stimulus selected is different from the one in (A). (D) Information contained in the LFP spectrum relative to naturalistic signals and averaged signals. Same color code as (C). Red dashed line same as (B).

First we changed the average rate of the signals leaving their spectral content unchanged. We added a constant value to each signal varying the parameter *B* in Equation 9. This corresponded to an increase of the average rate of the signal equal to *B* times the difference between its original average rate and the average rate across all signals. We will refer to the parameter *B* as ‘Baseline level’. In Figure 9A is shown the LFP spectrum for a single stimulus, averaged over 20 trials. When the baseline level was equal to 1, 2 and 3, the signal average value was 1.8, 1.9 and 2 spikes/ms, respectively. Low frequencies are not affected by changes in the baseline level, while the average modulation of the spectra in the 30–100 Hz range was 0.07, with a peak of 0.31 for 55 Hz. The average spectrum still resembled the one of the recorded data (Table 1). In Figure 9B we report the information about the naturalistic input carried by the LFP spectrum when the baselines are changed. When the differences among the average rates of the stimuli were increased, frequencies in the gamma band and above contained 0.1 more bits of information, while changes in the low frequency band were of 0.02 bits only. Both increasing and decreasing the baseline decreased the agreement of the information content of the spectrum of simulated and recorded LFPs, as measured by the reduced *χ* ^2^ (Table 1).

Second, we did the opposite: we changed the spectral content of the signals and left the average rate unchanged. Each signal was replaced with a constant function with a value equal to the average rate of the signal, therefore erasing all fluctuations. When this input was injected int the network, there was a decrease in the LFP power associated to low frequencies, but the rest of the spectrum did not display significant changes (Figure 9C), showing that signal oscillations determine only a narrow band of the signal output, while the rest is determined by noise and internal dynamics. The average information contained in the low frequency peak decreased from 0.11 from 0.02, below the significance level, while the one contained in the gamma band was left unchanged (Figure 9D).

### Effects of Changing Synaptic Strengths on Information Transfer

Finally, we investigated the effects of varying model parameters on the encoding properties of the network, by changing the values of the GABA and AMPA synaptic strength in equations 2–5. Default values used in the previous sections are displayed in Table 2 and new values are expressed as percentage of the default ones. GABA strength was modulated in the same way both in synapses projecting to interneurons and in those projecting to pyramidal neurons. AMPA strength was modulated in the same way in all AMPA synapses, both corticocortical and thalamocortical ones. These manipulations give extra insight on the differential role of excitatory and inhibitory synapses in determining the oscillations and process sensory information. Furthermore, some of the manipulations of synaptic parameters considered here are in principle reproducible experimentally using AMPA/GABA antagonists/agonists. They can therefore be considered as testable predictions of our model.

**Table 2:** Synaptic efficacies (mV).

	On Interneurons	On Pyramidal Neurons
GABA	2.7	1.7
Recurrent (‘cortical’) AMPA	0.7	0.42
External (‘thalamic’) AMPA	0.95	0.55

**Figure 10:**
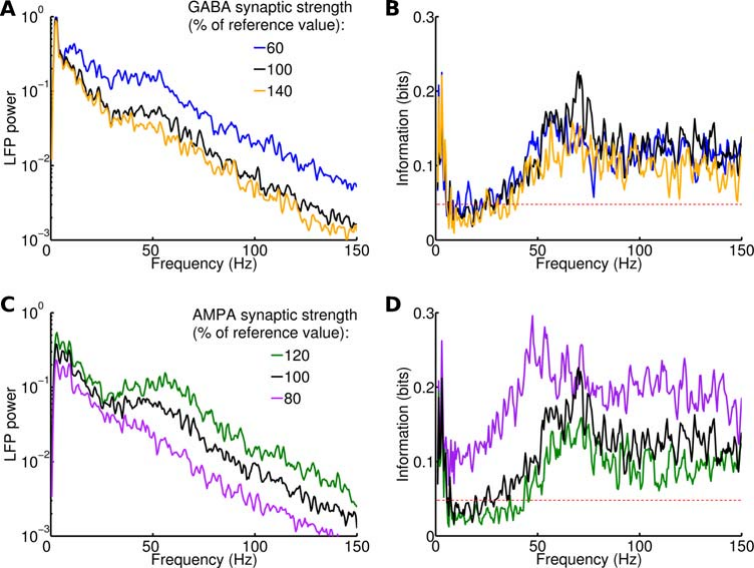
Effects of modulations in GABA and AMPA synaptic strength when naturalistic stimuli are injected. (A) Power spectrum of LFP for a single stimulus and different values of GABA strength, measured as percentage of the reference strength displayed in Table 2. Results are averaged over 20 trials. (B) Information contained in the LFP spectrum for the three synaptic strengths. Red dashed line corresponds to significance threshold (p<0.05; bootstrap test) for information. Same color code as (A). (C–D) Same as (A–B) for AMPA strength modulations.

Decreasing GABA strength led to an increase of the power associated to all frequencies (Figure 10A, Table 1). The increase was close to zero only for frequencies below 5 Hz, confirming that this range is largely determined by external modulation rather than internal dynamics. The relative increase of the power did not display any peak, suggesting that it could be simply due to the increase in average activity: the overall firing rate of the network increased of more than 50% when GABA was reduced to 60% of the default value. The information transfer was not affected significantly by changing GABA strength in the investigated range (Figure 10B, Table 1).

Increasing AMPA strength led to an increase of the corticocortical excitation but also of the input strength. An increase of 20% of AMPA strength was sufficient to increase the network firing rate of more than 40%. Increasing AMPA strength resulted in a general increase in the power associated to all frequencies (Figure 10C, Table 1). The increase was more pronounced for frequencies in the gamma band. On the other hand, increasing AMPA strength tended to decrease information transfer at high frequencies (Figure 10D, Table 1).

Overall, these simulations show that changing synaptic strengths affects quantitatively, but not qualitatively, our results. This means that our results are robust to parameter changes, provided the network stays in an inhibition-dominated regime in which individual neurons fire at low rates in an irregular fashion. Furthermore, these simulations provide an experimentally testable prediction: specific antagonists or agonists of synaptic transmission can affect the shape of the LFP spectrum without significantly changing its information content.

## Discussion

In recent years, the relationship between sensory stimuli and the temporal structure of LFPs has been the subject of extensive investigations (e.g., [8]–[10], [14], [45]–[47]). Since LFPs reflect integrative processes in areas such as the dendrite which are otherwise inaccessible, characterizing how LFPs encode sensory stimuli is crucial to understand how the microcircuitry of brain networks participates in sensation and shapes the magnitude and timing of local activity. Characterizing how LFPs encode information is also important to understand how neural signals can optimally communicate with brain-machine interfaces [48], and to better interpret the blood oxygenation level-dependent response, which correlates with several LFP bands [22],[49],[50]. Neurophysiological investigations have revealed that a broad range of LFP frequencies is involved in sensory processing, and that the dependence of LFPs on stimuli is complex. However, this complex dependence between the type of sensory stimuli and LFP frequency responses and its potential function has remained so far unexplained.

Here, we developed a theoretical framework for the understanding of the role of LFPs in sensory coding by studying the behavior of model networks of sparsely connected excitatory and inhibitory neurons that were stimulated dynamically. The interplay of excitation and inhibition captured by these networks is one fundamental feature of the organization of cortical microcircuit which is believed to shape the dynamics of local mass activation. Moreover, these networks intrinsically generate gamma-range oscillations, the most widely reported rhythm generated by sensory cortex. Building on the previous theoretical knowledge of how these networks generate oscillations when stimulated with time independent stimuli, we were able to provide several advances. First, we were able to quantify the information content of the fluctuations generated by the network and determine which LFP frequencies convey most information. Second, we found explicit coding rules between features of the stimulus dynamics and LFP frequency which are compatible with several neurophysiological reports. Third, we demonstrated that these coding rules lead to low and high LFP frequencies acting as largely independent information channels, in agreement with recent experimental data [14]. The significance of these findings and their relation to previous work will be discussed in detail next.

### Advances with Respect to Previous Modeling Work

Modeling the mechanisms of generation of oscillations in excitatory and inhibitory networks of spiking neurons is one of the most extensively studied topics in neural network dynamics. In this work, we reported several advances to the understanding of dynamics of recurrent networks. First, most previous model studies focused on the network dynamics under constant stimulation. We generalized these results to characterize the network dynamics to slowly-varying periodic and naturalistic stimuli. Second, rather than focusing only on the spectral structure of the network oscillations, we went a step further and quantified the information content of each band of the LFP spectrum in a way directly comparable to experimental findings. Combining a wide set of stimulations with the information theoretic analysis allowed us to derive simple and novel translation rules between stimuli and LFP responses.

Another advance in recurrent network modeling is that previous studies quantified the network output only as the total firing rate, whereas we quantified its output also in terms of simulated LFPs. This greatly facilitates the comparison with experimental recordings, permits a better validation of the models, and provides a mean to test explicitly some hypotheses on what LFPs reflect and how best to capture their properties with a simple model, which is itself an open question. We found that simulated LFPs based on sum of synaptic currents account for some of the main findings in stimulus encoding of LFPs: the modulation of the LFP gamma band when using stimuli eliciting firing rate modulations [3], [6]–[9], the entrainment of low frequency LFPs to stimulus oscillations [11],[16] and the way the two phenomena contribute to the information content of the whole LFP spectrum [14]. Therefore, despite LFPs potentially reflecting complex slow activity unrelated to synaptic activation such as voltage-dependent membrane oscillations [30] or spike afterpotentials [31], our study suggests that many coding properties of LFPs can be understood with simple models based on massed synaptic activation.

Previous modelling studies have computed local field potentials from detailed 3D models of networks of compartmental model neurons [32],[51],[52]. It would be interesting to investigate in such models how well the very simple LFP model introduced in the present paper correlates with the LFP model based on the detailed geometry of the underlying network. In particular, such a study could shed light on which combination of average AMPA/GABA currents best represents the ‘true’ LFP.

### Dependence of LFP Frequency on Stimulus

The main result of this study is the derivation of very simple rules of transformation between stimulus characteristics and the dynamics of the evoked LFP responses. Though very simple, these rules account for a large number of experimental observations. The first coding rule is that gamma-range LFPs carry information about sensory stimuli that provokes responses of neurons providing synaptic inputs to the specified area that vary from stimulus to stimulus only in terms of their total spike rate. This rule is in full agreement with the observation that stimuli of different contrast are encoded in V1 as gamma-range changes of LFPs [8], that direction of motion is encoded in area MT in the gamma-range LFPs [9], that orientation of gratings is encoded in V1 by gamma-range LFPs [7], and that sound frequencies are best encoded in the high frequencies of auditory field potentials [3], as all such stimuli elicit mostly changes of firing rate (rather than changes in the temporal response profile) in neurons providing synaptic inputs to the specified areas. As we discussed in Results, the simulations demonstrating this rule also correctly predict that the peak of maximal gamma power happens at a lower frequency than the peak of stimulus selectivity in the gamma range [7],[14]. The second coding rule is that stimulus-related changes of low-frequency cortical fluctuations encode information about slow dynamic features in the sensory or thalamic input that vary at the considered frequency. This is fully consistent with the finding that the low frequencies of LFPs can be entrained by slow periodic stimuli [11],[16], and that LFPs in V1 lock to some slowly varying dynamic features extracted from natural movies [14]. The double peak of information at low frequencies (<10 Hz) and in the gamma range (60–90 Hz) found in response to natural movies [14] can also be explained by this coding rule, since a natural movie contains both temporal frequency changes at low frequencies and objects and features capable of eliciting firing rate changes.

Our model was able to reproduce the most salient coding properties of LFPs in early visual cortex based on the hypothesis that the power modulations of LFPs at low frequencies followed temporal patterns emerging in the stimulus itself rather than being generated ex novo within the brain. This hypothesis stems from the observations that low frequency LFPs lock to slow rhythmic stimuli, and from the observations of [14] that the most informative component of the LFP power during movie stimulation was the stimulus modulation of the additional amount of power evoked during movie presentation with respect to spontaneous power. However, the brain is capable of internally generating rhythms in the low (≤10 Hz) frequency range through several cellular and network mechanisms [53] not implemented in our model. It is therefore conceivable that in many circumstances internal sources of slow rhythm generation are modulated by the external stimuli. In such cases, we would expect that additional stimulus-related information reflecting these internal processes may become available in the low frequency LFP modulations.

### Correlation between Stimulus Selectivity of Different Frequency Bands

It was recently reported that, during stimulation with naturalistic movies, low frequency LFPs and gamma-range LFPs in visual cortex are decoupled and act as independent information channels [14]. Our model was able to reproduce this finding and to provide an explanation. The independence between low frequency and gamma LFPs arises because they reflect two different input features (the slow frequency variation of the input rate and the total input spike count respectively) and these two input features appears to be largely independent when computed from LGN responses to natural movies (as demonstrated here).

One potential advantage of this frequency decomposition into independent transmission channels is that it may enable the cortical network to transmit more information by multiplexing it over several nested timescales [54]. Since LFPs reflect largely synaptic activity which may be partly decoupled from spiking activity, it is not guaranteed that all the information encoded in LFP oscillations may be used by other neural systems. The extent to which this information gain could be realized depends on how and whether the information carried by LFP oscillations can be read out by downstream systems. It seems plausible that the amount of gamma oscillations could be effectively read out by a downstream decoder, because gamma oscillations are often found to carry information redundant to that of spiking activity [14] and because gamma oscillations modulate transmission of signal across neural populations [55],[56]. Single cells in downstream networks could have intrinsic resonances at gamma frequencies allowing them to preferentially respond to such inputs [57]. Low frequency oscillations could be potentially read out as well, because these oscillations have greater spatial coherence and can thus be made more widely available to decoding networks. The phase or power of these oscillations may therefore be known to local target populations and it could be used to increase the information content of spikes by means of phase-of-firing or power-of-firing codes [15]. Whatever the extent to which this information may be used within cortex, we note that the independence of information carried by low and high frequency LFPs is potentially relevant to the practical development of brain machine interfaces, as it suggests that simultaneous decoding of different LFP bands may permit to obtain information which cannot be obtained by considering one frequency band only.

Our model did not only reproduce correctly the independence between low frequency LFPs and gamma LFPs, but reproduced well both signal and noise correlations over a wide range of LFPs frequencies. Notably, the only significant discrepancy between signal and noise correlation in real data [14] and in the present model, was that the model reported little or none noise correlations across all frequencies, whereas the real data presented strong noise correlation in the 12–24 Hz frequency range. These strong noise correlations were present also during spontaneous activity and were accompanied by little stimulus selectivity and little signal correlations during movie stimulation: Belitski and coworkers [14] hypothesized that the 12–24 Hz LFP frequency region related mainly to stimulus-independent neuromodulation. Since our model did not include any form of variation of neuromodulation across trials and independent from the stimulus, the fact that we could not find such noise correlations in the simulated data is compatible with their hypothesis that this phenomenon reflects the action of one or more neuromodulation pathway not specifically activated by the type of visual stimulus.

### Functional Characterization of LFP Bands

The analysis of EEGs and LFPs traditionally divides these measurements into a number of frequency bands, which correlate with distinct behavioral states and are thought to originate from different types of neural events triggered by different processing pathways such as sensory pathways or neuromodulation. However, the literature reports widely different, and often somehow arbitrary assumptions about which frequency range to investigate and how to set the boundaries of each band. A potential solution to this ambiguity is to set the boundaries ‘functionally’ [7], so as to extract as much information as possible about the stimuli. The coding rules obtained here suggest that, if this information theoretically optimal band partitioning is implemented, the optimal gamma range partitioning would remain roughly stable with stimuli and consistent with the one proposed in [7] because gamma range coding happens robustly whenever the network receives input rate modulations. On the other hand, partitioning the low frequency range into maximally informative bands may provide frequency boundaries that are dependent on the stimulus dynamics and not an intrinsic property of the network.

### Experimental Predictions Arising from the Model

An hypothesis of our model is that the stimulus-related changes in the power of low frequency LFPs follow at least in part the dynamics of the stimulus. For example, the high information content of low frequency LFPs found in response to natural movies was attributed to the temporal structure of the image flow, which contains the highest power and information in the low-frequency range. We suggest that a useful experimental paradigm that could help testing the hypotheses and coding rules presented here consists of changing the stimulus dynamics by using faster stimuli than natural movies and studying how this affects the informative LFP-frequency range. If the low frequency band modulations are mostly reproducing the modulations of the input spectrum, the exact position of the low frequency information peak should vary accordingly. Similarly, the interval in the gamma band containing information is predicted to depend on the rate range of the input, that for visual stimuli can be modulated with the image contrast. The study of the dependence of the information peaks on our model predicts also that, in the presence of GABA antagonists reducing but not blocking the inhibitory synaptic transmission, there is an increase of the power in the gamma band and only a very small decrease of the associated information. All of these predictions can be tested with current methodology, and the suggested experiments can help us understanding better the origin and function of the brain dynamics reflected in LFP fluctuations.

## Methods

### Model

The simulated network is composed of *N* = 5000 neurons. 80% of the neurons are taken to be excitatory, the remaining 20% are inhibitory [58]. The network is randomly connected: the connection probability between any directed pair of cells is 0.2 [59],[60]. Both pyramidal neurons and interneurons are described by leaky integrate and fire (LIF) dynamics [61]. Each neuron *k* is described by its membrane potential *V_k_* that evolves according to
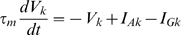
(1)where *τ_m_* is the membrane time constant (20 ms for excitatory neurons, 10 ms for inhibitory neurons, [62]), *I_Ak_* are the (AMPA-type) excitatory synaptic currents received by neuron *k*, while *I_Gk_* are the (GABA-type) inhibitory currents received by neuron *k*. Note that in Equation 1 we have taken the resting potential to be equal to zero. When the membrane potential crosses the threshold *V_thr_* (18 mV above resting potential) the neuron fires causing the following consequences: i) the neuron potential is reset at a value *V_res_* (11 mV above resting potential), ii) the neuron can not fire again for a refractory time *τ_rp_* (2 ms for excitatory neurons, 1 ms for inhibitory neurons).

Synaptic currents are the linear sum of contributions induced by single pre-synaptic spikes, which are described by a difference of exponentials. They can be obtained using auxiliary variables *x_Ak_*, *x_Gk_*. AMPA and GABA-type currents of neuron *k* are described by
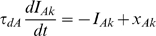
(2)

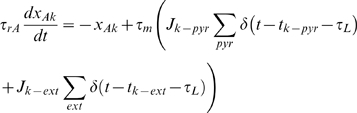
(3)

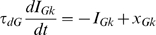
(4)

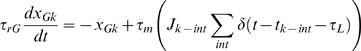
(5)where *t_k−pyr/int/ext_* is the time of the spikes received from pyramidal neurons/interneurons connected to neuron *k*, or from external inputs (see below). *τ_dA_* (*τ_dG_*) and *τ_rA_* (*τ_rG_*) are respectively the decay and rise time of the AMPA-type (GABA-type) synaptic current. *τ_L_* = 1 ms is the latency of post-synaptic currents. *J_k−pyr/int/ext_* is the efficacy of the connections from pyramidal neurons/interneurons/external inputs on the population of neurons to which *k* belongs. Most of the external input, i.e. all the signal and the largest part of the noise, is assumed in our model to come from the thalamus. The values of these parameters for all types of synapses are displayed in Tables 2 and 3. These values are of the order of magnitude of experimentally measured values [28], [63]–[66]. Modifying them affects quantitatively, but not qualitatively, our results, provided the network stays in an inhibition-dominated regime, as demonstrated in [26] and in the section *Effects of Changing Synaptic Strength on Information Transfer*. Changing parameters such as the latency *τ_L_* or the synaptic time constants can potentially change both location and shape of the peak in both LFP spectrum and information vs frequency curve. However, changing these parameters in the physiologically relevant range affects only mildly the agreement of the model with the data (see Table 4).

**Table 3:** Synaptic times (ms).

	*τ_L_*	*τ_r_*	*τ_d_*
GABA	1	0.25	5
AMPA on inter.	1	0.2	1
AMPA on pyr.	1	0.4	2

**Table 4:** Goodness-of-fit with experimental data of model LFP for different model parameters, measured with 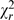 (see Methods).

	Spectrum Information Content	Average Spectrum
Model	0.7	1
*τ_L_* = 0.5 ms	1.1	1.0
*τ_L_* = 1.5 ms	0.9	0.8
*τ_rG_* = 0.5 ms	0.9	1.3
*τ_rG_* = 1 ms	1.2	1.7
*τ_dG_* = 4 ms	0.9	1.1
*τ_dG_* = 6 ms	0.8	1.7
*k* = 0.4 spikes/ms	3.1	2.1
*k* = 1.6 spikes/ms	3.5	67
*τ_n_* = 8 ms	0.8	1.4
*τ_n_* = 32 ms	1.0	1.0
*σ_n_* = 0.2 spikes/ms	1.0	2.0
*σ_n_* = 0.6 spikes/ms	1.4	4.3
LFP = 〈*V_m_*〉	0.8	10
LFP = |*I_A_*|+|*I_G_*|		
*α* = 0	0.7	1.3
*α* = 0.5	0.7	1.1
*α* = 1.5	0.8	1.0
*α* = 2	0.9	1.0

### External Inputs

Each neuron is receiving an external excitatory synaptic input (see previous section, last term in the r.h.s. of Equation 3). These synapses are activated by random Poisson spike trains, with a time-varying rate which is identical for all neurons. This rate is given by
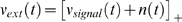
(6)where *ν_signal_*(*t*) represents the signal, and *n*(*t*) is the noise. […]. is a threshold-linear function, [*x*]_+_ = *x* if *x*>0, [*x*]_+_ = 0 otherwise, to avoid negative rates which could arise due to the noise term. Each simulation is repeated 20 times with the same signal and a noise generated independently for each simulation. A single run is called a *trial*. We now describe signal and noise separately.

### Signal

We use three types of signals: constant; periodic; and ‘naturalistic’. All signals last 2 seconds.

- The **constant signals** used in the section *How gamma oscillations are modulated by the firing rate of the input stimulus* are defined by
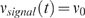
(7)where *ν*_0_ is a constant rate, with a value ranging from 1.2 to 2.6 spikes/ms.
- The **periodic signals** used in the section *Response of the network to low frequency oscillatory inputs* are defined by
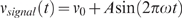
(8)where *ν*_0_ is a constant baseline equal to 1.6 spikes/ms, *A* is the amplitude of the oscillatory component, and *ω* is the frequency. The latter were varied in different simulations respectively from 0.4 to 1.6 spikes/ms and from 4 to 16 Hz.
- **‘Naturalistic’ signals** were built from a single electrode MUA recording from LGN of anesthetized monkey watching natural movie scenes. This MUA was measured as the absolute value of the high pass filtered (400–3000 Hz) extracellular signal recorded from an electrode placed in the LGN while the monkey was presented binocularly a color movie (we refer to [67] for full details on experimental methods). The MUA measured in this way is thought to represent a weighted average of the extracellular spikes of all neurons within a sphere of ≈140–300 µm around the tip of the electrode [29], and thus gives a good idea of the spike rate fluctuations of a patch of geniculate input to cortex during viewing of natural stimuli. We took 40 consecutive seconds of LGN MUA recordings during movie presentation, we divided it into 20 non-overlapping intervals of 2 seconds (ideally corresponding to different movie scenes) following the procedure used in [14], and each interval was considered as a different visual stimulus.

The standard high-pass filtering and rectification procedure that we used provides a MUA signal that correlates well with the power of the local spiking activity measured e.g. by detecting spike times of the closest neurons with a threshold crossing criterion [29]. However, the high-pass filtering and the rectification are likely to flatten out differences of the average spike rate between different stimuli with respect to the true underlying spike rate. This is because the rectification reduces the dynamic range and the high pass filtering may preserve some small amount of low frequency noise that end up as spike rate to all stimuli. To compensate for this, we amplified the differences across stimuli of MUA rate by building a signal *Q_i_*(*t*):
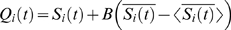
(9)where *S_i_*(*t*) is the original time series of the stimulus *i* recorded in the LGN, 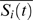 is the average value of the stimulus, and 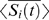 is the average value of the whole recording. This manipulation leaves power of frequencies >0.5 Hz unchanged. To set the value of *B*, we used the following procedure. We know from simulations with constant inputs that changes in input rate translate into modulations in the output gamma power band of the LFP. We computed then the coefficient of variation (CV) of the gamma power (sum of the power of frequencies in between 30 and 100 Hz) among the different stimuli for the LFP recording from V1. We selected recordings from the same monkey, experiment and movie screening of the MUA recording from LGN we were considering. The resulting CV value, averaged over 7 electrodes, was CV = 0.26±0.05. Increasing *B* in Equation 9 leads to an increase in the gamma band LFP CV. We set *B* = 2, for which CV = 0.24, consistent with the data. In Figure 9, we use *B* = 1 (corresponding to CV = 0.18) and *B* = 3 (CV = 3) to investigate the sensitivity of the dynamics to changes in parameters defining external stimuli.

The MUA computed in this way was expressed in mV and needed to be converted into spike rates units to be fed to the network. A series of papers [68]–[70], report similar estimates of ∼6 spikes/s for the activity of LGN neurons in the absence of visual stimulations. Anatomical studies estimate that about 130 LGN synapses project to each V1 neuron in the macaque [71],[72]. Multiplying these two numbers we estimated an average baseline of 0.8 spikes/ms reaching V1 from LGN. The same set of papers [68]–[70], shows that during movie stimulations the firing rate of LGN neurons is ∼12 spikes/s, so we set the average value of the signal to be 1.6 spikes/ms. The final equation determining the naturalistic signal is then:
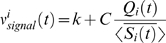
(10)where *k* = 0.8 spikes/ms, and *C* = 0.8 spikes/ms.

### Noise

There are two sources of noise in our model. The first is due to the fact that *n*(*t*) in Equation 6 is a stochastic variable, generated according to an Ornstein-Uhlenbeck process,
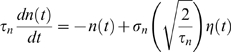
(11)where *σ_n_* is the standard deviation of the noise, and *η*(*t*) is a Gaussian white noise. The mean value of this process is zero, and its power spectrum is flat up to a cut-off frequency, 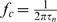 and then decays as *f* ^−2^. The time constant *τ_n_* was set to 16 ms to have *f_c_* = 10 Hz, and the standard deviation *σ_n_* was set to 0.4 spikes/ms based on CV values for LGN activity [70].

The second source of noise is due to the fact that different neurons receive independent realizations of a Poisson process, with the same time-varying rate *n*(*t*).

### Input Parameters

Signal parameters *k*, *C* and the noise parameter *σ_n_* have been set to be compatible with experimentally inferred values of thalamic activity during the screening of natural movies [68]–[70], *B* to be compatible with gamma oscillation range in V1 recordings in the same conditions, while we have chosen *τ_n_* so that the noise is maximum for frequencies lower than 10 Hz, because spontaneous activity in the visual cortex acts mainly on this timescale [73],[74]. We tested the robustness of our results to changes in these values. As an example, a key result of the present work is the presence of two peaks in the information content of the spectrum, for low frequencies and in the gamma band, when the network is presented with naturalistic stimuli (Figure 7B). Figure 9C shows that modulations of *B* led to changes in the information content of the gamma band and higher frequencies, but do not affect low frequencies content and the shape of the Information(frequency) function. Figure S3 shows the robustness of our conclusion to variations in the input parameters described in the previous paragraphs.

- Figure S3A: when *k* was varied by a factor of 4, the low frequency peak remained unchanged. The gamma peak was always present, but as *k* was increased, the peak was set at higher frequencies, since the overall input strength increased.
- Figure S3B: when *τ_n_* was increased by a factor of 4, the information content in the gamma band remained unchanged, while the low frequency peak decreased in height (the overall level of noise remained the same, but the larger *τ_n_* the more it was focused on low frequencies) but remained at 2 Hz. This means that results are robust to variations in the cutoff frequency from 5 to 20 Hz.
- Figure S3C: when *σ_n_* was varied of a factor of 3, the total information contained in the gamma peak changed by less than 50% and its shape remained stable. The low frequency peak was always set at 2 Hz, but its height varied by a factor of ∼5.

Table 4 summarizes the effects of changing these parameters on the agreement between model and data, as measured by the reduced *χ* ^2^. It shows that this agreement is fairly robust to changes in *τ_n_*, while both increasing and decreasing *σ_n_* and *k* deteriorates this agreement.

### Numerical Methods

Simulations were done with a Runge-Kutta algorithm with time step Δ*t*. For equations (1–5) Δ*t* = 0.05 ms. Since the experimental recording frequency is 500 Hz, the input to the network (included the noise) was updated every 2 ms.

### Generation of Simulated Local Field Potentials

LFP is a common measure of neuronal activity, but it is still not completely clear how the LFP is related to single neuron variables like synaptic or ionic currents, and membrane potentials. Computational models sometimes use as a description of the LFP the average membrane potential of the neurons of the network [75], even though it seems definitely more likely that the LFP is rather more directly related to the synaptic activity [29]. The spectrum of the average membrane potential in our model has a faster decay at high frequencies than the measured LFP, and therefore does not reproduce it well (Figure S4). However, the information content of the average membrane potential turns out to be similar to the one of the recorded LFPs (Table 4).

On the opposite side, LFPs have been computed using compartmental neuron models [32],[51]. The model used in [51] adopted the neuronal structure described in [76]: dendritic branches were divided into cilindrical compartments of 50 µm length. Each compartment contained many synapses, whose characteristics depended on the branch (apical, basal etc). The LFP was computed for every point in the space surrounding the neuron as the total extracellular potential originated by the trasmembrane currents of the hundreds of different compartments. In [32] the procedure was similar but the neuronal structure was reduced to a total of 15 compartments. In both models, LFPs were originated by synaptic currents on pyramidal neurons dendrites.

Here, we resorted to a similar but simpler approach, which takes into account that our model makes no attempt to replicate the spatial organization of cortical neurons, and thus the sum in space of currents has to be abstracted and simplified, as follows. To capture in a simple way the fact that pyramidal cells contribute the most to LFP generation because their apical dendrites are arranged in an approximate open field configuration, we assumed that the LFP is generated by the dipole-like dendrites of pyramidal cells, in which currents flow in the cell through apical excitatory synaptic contacts while they flow out through basal inhibitory contacts [77]. This suggests to model LFPs as the sum of the absolute values of AMPA and GABA currents (|*I_A_*|+|*I_G_*|) on pyramidal cells, which was the model we adopted in this work, and that was able to reproduce correctly both the power spectrum of recorded LFPs and its information content (Table 4, Figure 7B, and Figure S4). Taking the LFP to be a different linear combination of AMPA and GABA currents give rise to qualitatively similar results (Table 4 and Figure S4). LFP signals are high-passed at 1 Hz with a 4th order Butterworth filter to reproduce experimental recording procedures of [14].

### Spectral Analysis

The power spectrum in each trial obtained in response to each simulated stimulus was obtained using the multitaper technique [78], which provides an efficient way to simultaneously control the bias and variance of spectral estimation by using multiple Slepian data tapers and was the one mostly used in recent neurophysiological studies of LFPs [8],[14]. The use of Slepian functions minimizes the bias, whereas the use of multiple orthogonal tapers on the same data minimizes the variance. The Slepian functions are defined in terms of their length *L* in time and their bandwidth *W* in frequency. For each choice of *L* and *W*, up to *K* = 2*LW*−1 tapers are highly concentrated in frequency, having 90% of their power within the interval [−*W*, *W*], and can be averaged for spectral estimation. To reduce the spectral bias, the average over tapers was computed using the adaptive procedure described by [78]. A simplified way of conceptualizing the multitaper method is that it provides an average over the local frequency ensemble with a range 2*W* [78]. The value of *W* should be chosen on the basis of empirical considerations. Here, we chose *LW* = 2 because it matches the one used by [14] and thus makes the comparison between simualtion and experiments more transparent.

For the sake of the entrainment analysis only (Figure 6D and Figure S1), both the LFP and the naturalistic stimuli were band-passed at selected frequencies with a Kaiser window with a 2 Hz bandwidth, very small passband ripple (0.01 dB), and high stopband attenuation (60 dB) [14]. Forward and backward filtering was used to eliminate phase shifts introduced by the filter. For each band-passed signal the phase was extracted by the means of the Hilbert transform. The phase of each band-passed LFP was compared with the input signal phase, for periodic signals, or with the phase of the band-passed naturalistic signal. We computed then the circular variance [79] of the input-output phase difference 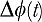 as 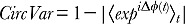. Circular variance ranges from 0 (perfectly locked phases) to 1 (random phase differences uniformly spread over the circle).

### Measures of Information Carried by the Neural Response Power

To determine how well the power of LFPs *r_f_* at a certain frequency *f* encodes the stimuli, we computed the mutual information *I*(*S*, *R_f_*) between the power *r_f_* at frequency *f* and the stimuli *S* [80], as follows:
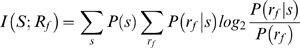
(12)where P(s) is the probability of the presentation of the stimulus *s*, *P*(*r_f_*) the probability of the frequency *f* to have power *r_f_* over all trials and all stimuli, *P*(*r_f_*|*s*) the probability of *r_f_* to be observed when stimulus *s* is presented. The above single-frequency information analysis can be extended to compute how much information about the stimulus we can obtain when combining together the power *r_f_*_1_ and *r_f_*_2_ at two different frequencies. The mutual information that the joint knowledge of the powers *r_f_*_1_ and *r_f_*_2_ conveys about the stimulus is as follows:
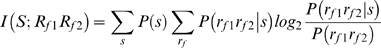
(13)If two frequencies were tuned to completely different stimulus features, and they did not share any source of noise, then we would expect *I*(*S*; *R_f_*_1_ *R_f_*_2_) to be equal to the sum of the information that each frequency conveys separately. It is therefore useful to introduce the following “information redundancy” [41],[81],[82]:
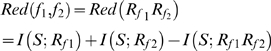
(14)When redundancy is positive, the two frequencies are said to convey redundant information about the stimulus; when redundancy is zero, the two frequencies are said to convey independent information.

Estimates of mutual information often suffer from the limited amount of data available to calculate the conditional probabilities and the resulting statistical errors. These errors translate into a bias in the information estimate. To correct for this bias, we implemented a multi-step procedure, which follows the ideas presented in [15],[83] and was used, described and tested in our previous studies [14],[15]. In brief, the bias estimation of the information contained in the multidimensional responses was greatly reduced at the very source, and made negative, by using the “shuffling” technique described in [83]. Then, a well-established quadratic extrapolation procedure [84] was used to further reduce the bias. We finally evaluated and subtracted out any (small in this dataset) residual bias by the “bootstrap” procedure fully reported in [15]. This procedure provides information estimates which are very accurate. The performance of these procedures on simulated data has been reported previously [15],[83]. In particular, when tested on data with statistics similar to the one considered here and with number of trials similar to the one available here, the resulting information estimates are very tight and present a very small residual error in the estimate of the bias.

### Quantification of Signal and Noise Correlation

To determine which frequencies have related stimulus selectivity and which have shared sources of variability, we performed a linear analysis of correlations across frequencies of both the signal and the noise, as follows. The correlation of the mean responses across different stimuli of two frequencies are called “signal correlations” [40],[41],[85] because they are entirely attributable to stimulus selectivity. The signal correlation coefficient was computed, for each frequency pair and channel, as the Pearson correlation across stimuli of the trial-averaged responses. Positive values indicate that the two frequencies have similar stimulus preference, whereas a zero values indicates that the two frequencies prefer totally uncorrelated stimuli. Correlations manifested as covariations of the trial-by-trial fluctuation around the mean response to the stimulus are traditionally called “noise correlations” [40],[85]. Since these noise covariations are measured at fixed stimulus, they ignore all effects attributable to shared stimulation. To quantify the strength of noise correlations, we computed the Pearson correlation coefficient (across trials at fixed stimulus) of the trial-average-subtracted powers *r_f_*_1_ and *r_f_*_2_, and then we averaged it over all stimulus windows. This quantifies the correlations of the variations around the mean at each trial and stimulus window. Positive values of noise correlation means that when the power of one frequency fluctuates over its mean values, the power in the other frequency is also more likely to do so.

### Goodness of Fit Measurements

For every set of parameters shown in Tables 1 and 4, the information content of the spectrum of the simulated LFP was compared with the information contained in the spectrum of the real LFPs recorded with seven electrodes in V1 synchronously to the LGN recording used to construct the naturalistic signals. *I*(*S*, *R_f_*) is the information about the set of stimuli contained in the power of the frequency *f* of the simulated LFP. We computed the information associated to the power of each frequency also for the LFP recorded in every electrode and then we computed its mean (I*_V_*_1_(*f*)) and its standard deviation (σ*_V_*_1_(*f*)) across the electrodes. The measure used to quantify the agreement between the model and the data was the reduced chi squared 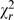:
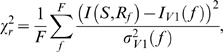
(15)using a total number of frequencies *F* equal to 400 (from 0.5 to 200 Hz in a 0.5 Hz step). A value of 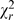 close to 1 suggest that the model is as different from the data as the data are different among different electrodes. The same procedure was then applied to compare the model LFP spectrum and the recorded LFPs spectra, both averaged over all scenes and trials.

Other methods, such as Dynamic Expectation Maximization [86],[87] or Kalman Filtering [88] could be used to obtain a more principled measure of correspondence between model and data, the best fit parameters and the parameter confidence. However, these more sophisticated procedures were in practice not applicable to our simulations because of the high dimensional parameter space and because of the long time taken to run the analysis (6 hours per parameter setting on our workstation). For this reason, we resorted to fix most parameters from plausible literature values, and then tune them by hand to obtain a good fit as measured by 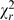. The robustness to parameter variations was empirically determined by starting from the so-determined optimal parameters and checking for biologically plausible values the reduced 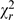 of both information and power spectrum.

## Supporting Information

Figure S1 Signal-LFP entrainment for different amplitudes of periodic stimuli. Circular variance of the phase difference between periodic input signals of different frequencies and the LFP bandpassed at corresponding frequencies when the signal amplitude is 0.4 spikes/ms (A) and 1.6 spikes/ms (B). The entrainment is inversely proportional to the value of the circular variance
(0.28 MB TIF)

Figure S2 Frequency correlations across naturalistic stimuli. (A) Joint information and (B) Redundancy for frequency pairs.
(0.57 MB TIF)

Figure S3 Effects of input parameters modulations on information content of LFP when naturalistic stimuli are injected. In all panels the black line corresponds to the combination of parameters value used in the Results sections, and the red dashed line to significance threshold (p<0.05; bootstrap test). (A) Information associated to each frequency when parameter k in Equation 10, corresponding to the signal baseline, was varied. (B) Same as (A) when parameter τ_n_ in Equation 11 was varied. The different values correspond to a stronger noise in the range 0–5 Hz (green line), 0–10 Hz (black line), 0–20 Hz (blue line). (C) Same as (A) when parameter σ_n_ in Equation 11, describing the amplitude of noise fluctuations, was varied.
(0.94 MB TIF)

Figure S4 LFP Models. (A) Comparison of spectra of LFP recorded in V1 of anesthetized monkey watching natural movie scenes and spectra of different LFP models when the network was injected with naturalistic signals based on LGN activity recorded in the same experiments. Gray area represents the mean±std range of power across 7 different electrodes recording synchronously from different sites. The colored lines are spectra obtained with the following LFP models: average membrane potential (green line), sum of absolute value of AMPA currents on pyramidal neurons (pink), sum of absolute values of AMPA and GABA currents on pyramidal neurons (black), and the same sum with a weight 2 assigned to GABA currents (orange). Spectra are averaged over all trials and scenes. (B) Comparison of information content of the spectrum of recorded and simulated LFPs. Same data sets and color code as (A). Red dashed horizontal line indicates significance threshold (p<0.05, bootstrap test).
(0.62 MB TIF)
