# Supplementary figures and images for: Encoding of Naturalistic Stimuli by Local Field Potential Spectra in Networks of Excitatory and Inhibitory Neurons
Source: PLoS Comput Biol. 2008 Dec 12;4(12):e1000239. doi: 10.1371/journal.pcbi.1000239 (PMC2585056; doi:10.1371/journal.pcbi.1000239)

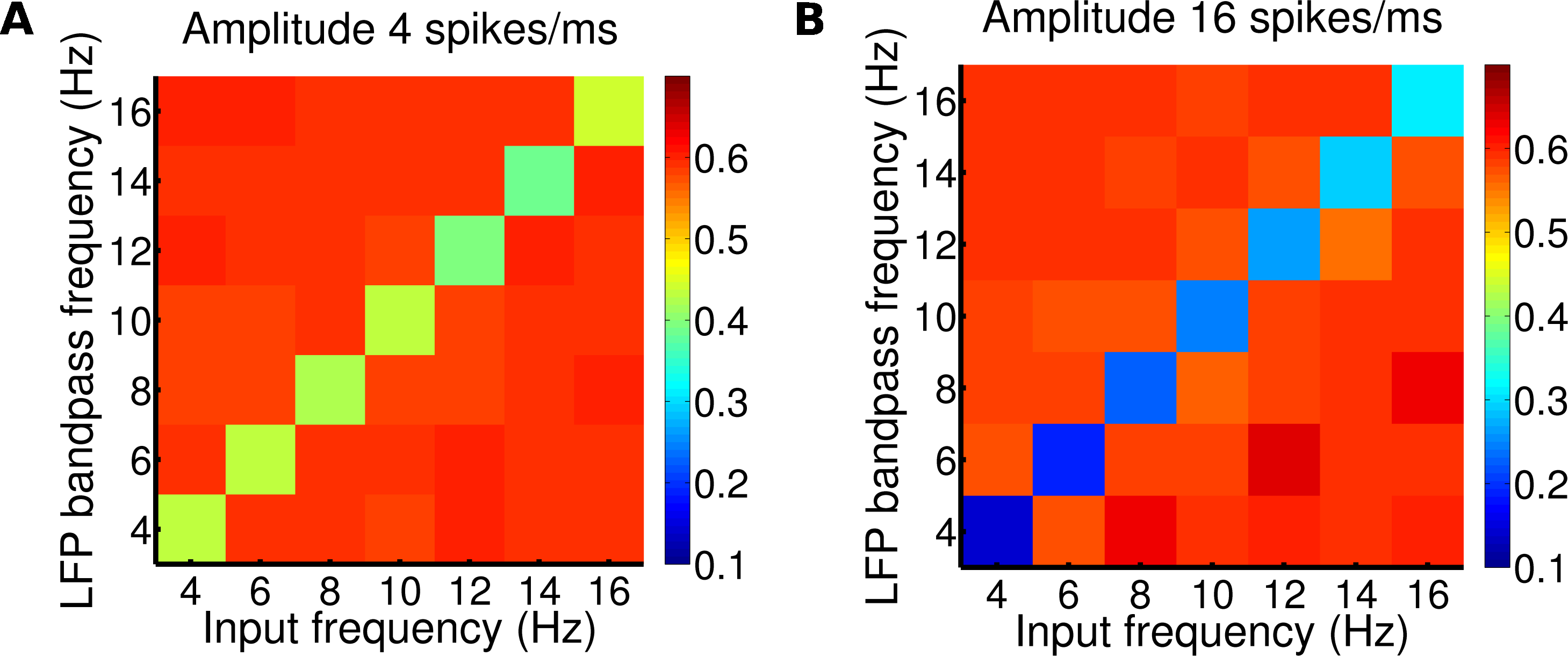

Supplement: Figure S1 — Signal-LFP entrainment for different amplitudes of periodic stimuli. Circular variance of the phase difference between periodic input signals of different frequencies and the LFP bandpassed at corresponding frequencies when the signal amplitude is 0.4 spikes/ms (A) and 1.6 spikes/ms (B). The entrainment is inversely proportional to the value of the circular variance (0.28 MB TIF) [file pcbi.1000239.s001.tif]

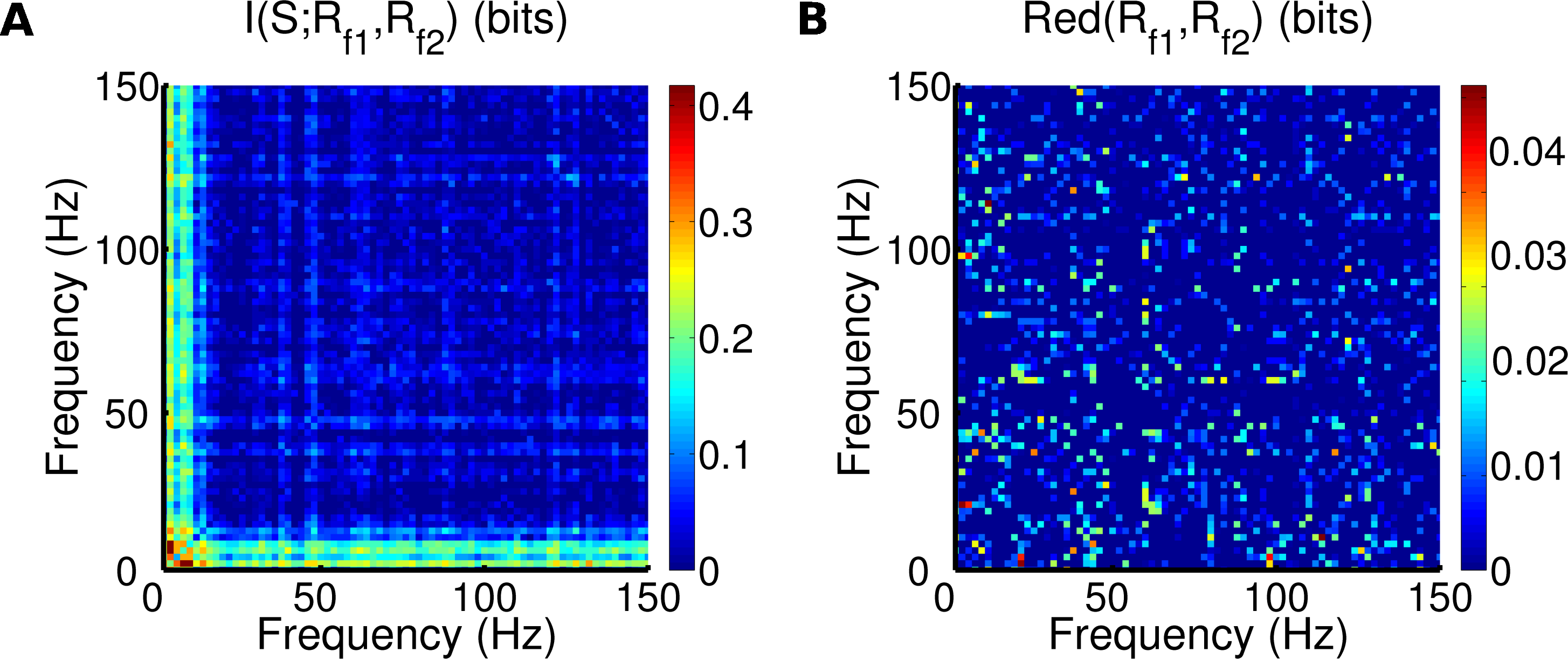

Supplement: Figure S2 — Frequency correlations across naturalistic stimuli. (A) Joint information and (B) Redundancy for frequency pairs. (0.57 MB TIF) [file pcbi.1000239.s002.tif]

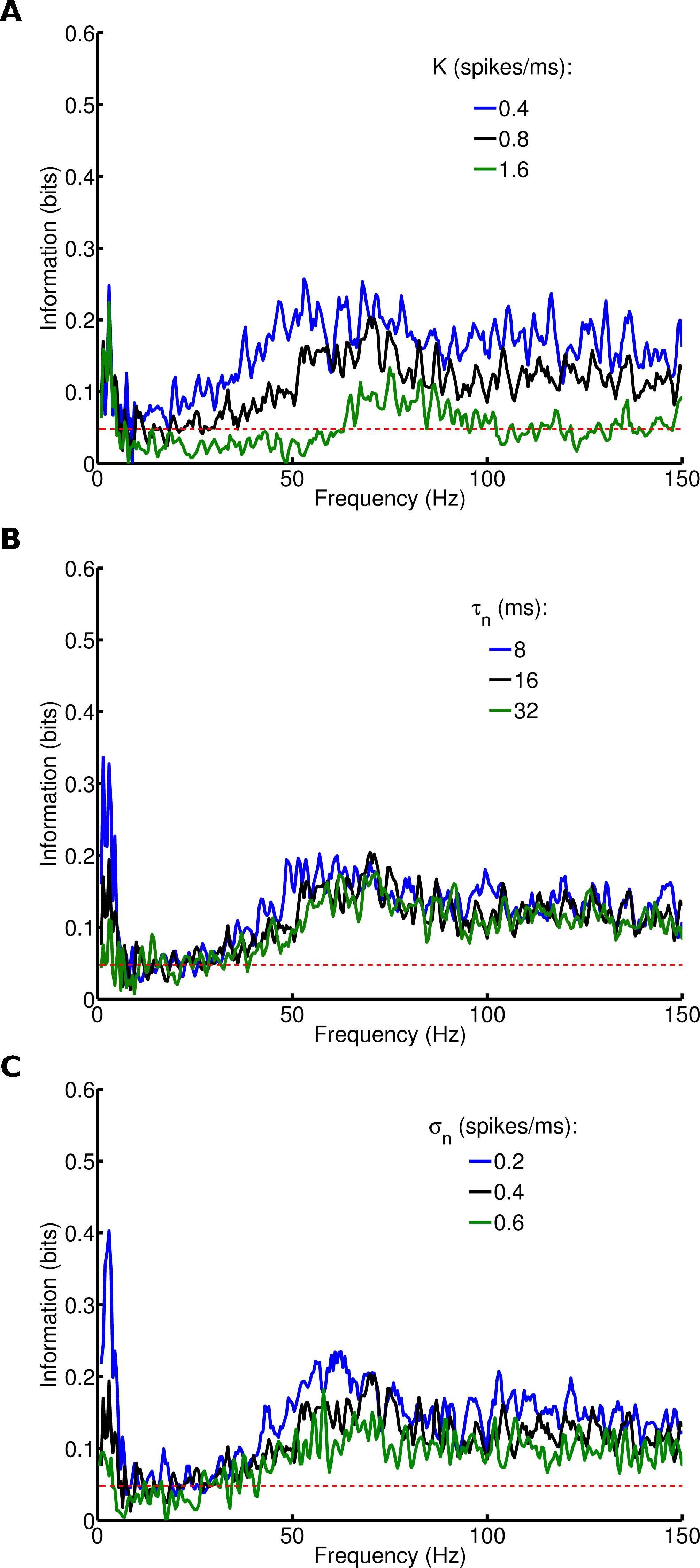

Supplement: Figure S3 — Effects of input parameters modulations on information content of LFP when naturalistic stimuli are injected. In all panels the black line corresponds to the combination of parameters value used in the Results sections, and the red dashed line to significance threshold (p<0.05; bootstrap test). (A) Information associated to each frequency when parameter k in Equation 10, corresponding to the signal baseline, was varied. (B) Same as (A) when parameter τn in Equation 11 was varied. The different values correspond to a stronger noise in the range 0–5 Hz (green line), 0–10 Hz (black line), 0–20 Hz (blue line). (C) Same as (A) when parameter σn in Equation 11, describing the amplitude of noise fluctuations, was varied. (0.94 MB TIF) [file pcbi.1000239.s003.tif]

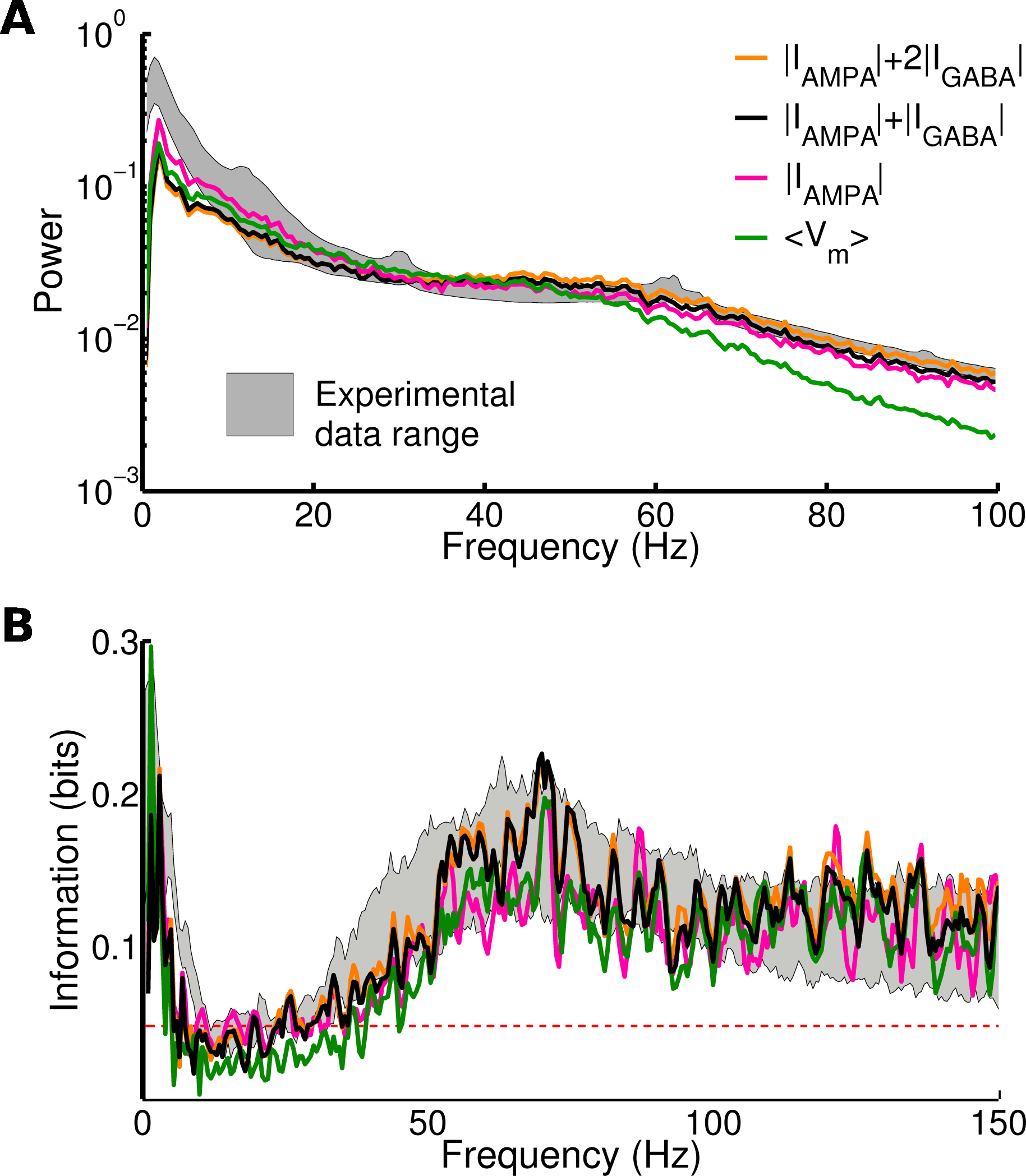

Supplement: Figure S4 — LFP Models. (A) Comparison of spectra of LFP recorded in V1 of anesthetized monkey watching natural movie scenes and spectra of different LFP models when the network was injected with naturalistic signals based on LGN activity recorded in the same experiments. Gray area represents the mean±std range of power across 7 different electrodes recording synchronously from different sites. The colored lines are spectra obtained with the following LFP models: average membrane potential (green line), sum of absolute value of AMPA currents on pyramidal neurons (pink), sum of absolute values of AMPA and GABA currents on pyramidal neurons (black), and the same sum with a weight 2 assigned to GABA currents (orange). Spectra are averaged over all trials and scenes. (B) Comparison of information content of the spectrum of recorded and simulated LFPs. Same data sets and color code as (A). Red dashed horizontal line indicates significance threshold (p<0.05, bootstrap test). (0.62 MB TIF) [file pcbi.1000239.s004.tif]
